# Recommendations for cellular and molecular pathology input into clinical trials: a systematic review and meta‐aggregation

**DOI:** 10.1002/cjp2.199

**Published:** 2021-02-26

**Authors:** Shujing Jane Lim, Kurinchi Gurusamy, Daniel O'Connor, Abeer M Shaaban, Daniel Brierley, Ian Lewis, David Harrison, Timothy James Kendall, Max Robinson

**Affiliations:** ^1^ Department of Cellular Pathology Newcastle upon Tyne Hospitals NHS Foundation Trust Newcastle Upon Tyne UK; ^2^ Division of Surgery and Interventional Sciences University College London London UK; ^3^ The Medicines and Healthcare Products Regulatory Agency London UK; ^4^ Department of Histopathology Queen Elizabeth Hospital Birmingham Birmingham UK; ^5^ Institute of Cancer and Genomic Sciences University of Birmingham Birmingham UK; ^6^ Unit of Oral and Maxillofacial Pathology University of Sheffield Sheffield UK; ^7^ National Cancer Research Institute London UK; ^8^ University of St Andrews St Andrews UK; ^9^ University of Edinburgh Centre for Inflammation Research, University of Edinburgh Edinburgh UK

**Keywords:** clinical trial, pathology, systematic review, checklist, guideline, protocol, recommendations, SPIRIT

## Abstract

The SPIRIT (Standard Protocol Items: Recommendations for Interventional Trials) 2013 Statement was developed to provide guidance for inclusion of key methodological components in clinical trial protocols. However, these standards do not include guidance specific to pathology input in clinical trials. This systematic review aims to synthesise existing recommendations specific to pathology practice in clinical trials for implementation in trial protocol design. Articles were identified from database searches and deemed eligible for inclusion if they contained: (1) guidance and/or a checklist, which was (2) pathology‐related, with (3) content relevant to clinical trial protocols or could influence a clinical trial protocol design from a pathology perspective and (4) were published in 1996 or later. The quality of individual papers was assessed using the AGREE‐GRS (Appraisal of Guidelines for REsearch & Evaluation – Global Rating Scale) tool, and the confidence in cumulative evidence was evaluated using the GRADE‐CERQual (Grading of Recommendations Assessment, Development and Evaluation–Confidence in Evidence from Reviews of Qualitative research) approach. Extracted recommendations were synthesised using the best fit framework method, which includes thematic analysis followed by a meta‐aggregative approach to synthesis within the framework. Of the 10 184 records screened and 199 full‐text articles reviewed, only 40 guidance resources met the eligibility criteria for inclusion. Recommendations extracted from 22 guidance documents were generalisable enough for data synthesis. Seven recommendation statements were synthesised as follows: (1) multidisciplinary collaboration in trial design with early involvement of pathologists, particularly with respect to the use of biospecimens and associated biomarker/analytical assays and in the evaluation of pathology‐related parameters; (2) funding and training for personnel undertaking trial work; (3) selection of an accredited laboratory with suitable facilities to undertake scheduled work; (4) quality assurance of pathology‐related parameters; (5) transparent reporting of pathology‐related parameters; (6) policies regarding informatics and tracking biospecimens across trial sites; and (7) informed consent for specimen collection and retention for future research.

## Introduction

Randomised controlled trials are considered the ‘gold standard’ in medical research for comparing the safety and effectiveness of novel interventions as they minimise bias compared to other empirical study designs, thus delivering a high level of evidence [1, 2]. The confidence in the quality of evidence drawn from the conclusions of clinical trials is ultimately only as good as the robustness of the study design and methodology, which encompasses the quality assurance of detailed processes involved in the delivery of the trial [2, 3]. Poorly planned clinical trials result in misleading findings from suboptimal trial execution and analysis, thus wasting resources and potentially harming patients [4]. Such issues can be mitigated by methodologically rigorous clinical trial protocols to facilitate the design, conduct, analysis, and reporting of reliable clinical trials [3, 4, 5]. For this reason, the SPIRIT (Standard Protocol Items: Recommendations for Interventional Trials) 2013 Statement was developed to provide standardised guidance for inclusion of key methodological components in clinical trial protocols [6]. However, these standards do not include guidance specific to pathology input in clinical trials.

To attain methodological rigour in trials, there is increasing recognition of the need for pathologists to be involved early in trial planning and design [7, 8, 9, 10]. Current literature contains a considerable number of reviews and perspective papers offering opinions from various pathologists and biomedical scientists on different specific aspects of the laboratory workflow that could improve clinical trial quality [7, 8, 9, 10, 11, 12, 13, 14, 15, 16, 17, 18, 19, 20, 21, 22, 23, 24, 25, 26, 27, 28, 29, 30, 31, 32, 33, 34, 35, 36, 37, 38, 39, 40, 41, 42]. However, there is no single comprehensive guidance document covering all aspects of pathology workflow feeding into various stages of the clinical trial process. Furthermore, trialists writing protocols typically have a limited understanding of the role of the laboratory and pathologist in their studies and may overlook key issues that need to be addressed during the design phase.

### Aims and objectives

This systematic review aims to synthesise existing guidance and recommendations for pathology practice in clinical trials for implementation in trial protocol design.

Based on recent systematic review typology recommendations by Munn *et al* [43] from the Joanna Briggs Institute (JBI), this is a systematic review of expert opinion. The review question was therefore developed based on the recommended PICo (*P*opulation, phenomena of *I*nterest, *Co*ntext) framework, as follows:

- P (*P*opulation): individuals involved in the design, conduct, and analysis of clinical trials requiring pathology input.
- I (phenomena of *I*nterest): recommendations or guidelines specific to pathology input (within the ‘context’ below).
- Co (*Co*ntext): the entire clinical trial process, from the design and conduct of the trial to the analysis and dissemination of trial findings.

Review question: What recommendations are available to guide cellular and molecular pathology input in clinical trials?

## Materials and methods

### Protocol and registration

The protocol was prospectively registered on the Open Science Framework online repository (Registration DOI: https://doi.org/10.17605/osf.io/jeqtx) [44]. This review was reported in accordance with the 2009 PRISMA (Preferred Reporting Items for Systematic Reviews and Meta‐Analyses) statement [45].

### Eligibility criteria

Resources were deemed eligible for inclusion if they contained: (1) guidance (in the form of guidelines or expert recommendations) and/or a checklist, which is (2) pathology‐related, with (3) content relevant to clinical trial protocols or could influence a clinical trial protocol design from a pathology perspective. In addition, the year of publication was restricted to include resources published in 1996 or later, after the publication of the CONSORT (Consolidated Standards Of Reporting Trials) Statement guidelines [46]. There were no other restrictions by language or publication type.

### Information sources and search strategy

The search strategy was devised by initially scoping the literature on MEDLINE and EMBASE via the Ovid platform, as well as an internet search on Google Scholar to identify a comprehensive set of relevant search terms. The full search strategies for all databases and web searches are available in the review protocol [44]. Free‐text terms such as ‘(histolo*; OR patholo*)’ AND ‘(checklist; OR guideline; OR recommendation)’ AND ‘(clinical trial; OR protocol)’, along with equivalent controlled vocabulary terms, were used in the search of the MEDLINE (Ovid), EMBASE (Ovid), and Cochrane Library databases. Additional search terms such as ‘biomarker*’, ‘molecular diagnos*’, ‘practice guid*’, and ‘study design’ were also applied across the MEDLINE and EMBASE databases. Web searches on Google and Google Scholar were performed using the advanced search function with the keywords ‘(Pathology; OR Histology; OR Biomarkers)’ AND ‘(Guideline; OR Checklist)’ AND ‘Clinical trial’. Only the first three pages (30 results) from each internet search were screened as lower‐ranked results are less relevant to the search query [47]. The databases and search engines were searched from 1 January 1996 to 13 January 2020. Besides the year of publication, no other limitations (such as language restrictions or restrictions by publication status) were placed on the searches.

### Selection of guidance resources

All citations were imported into Mendeley Desktop software (Elsevier, London, UK; Version 1.19.5/2019). The titles and abstracts of the records were screened by two reviewers; SJL screened all the records, and TJK and MR acted as second reviewers. Inter‐rater agreement between the two reviewers was measured using Fleiss' kappa (*κ*), and the strength of agreement was interpreted according to Landis and Koch [48]. In cases where an abstract was not available, the full text of the article was retrieved. Resources that both reviewers selected for inclusion were subjected to full‐text review, while any disagreements were arbitrated by a third reviewer (TJK or MR). Two reviewers independently reviewed the full texts; any disagreements were discussed with a third reviewer and resolved by consensus. Resources not meeting the inclusion criteria upon review of full texts were excluded with reasons provided.

### Quality assessment of guidance resources

Each eligible guidance resource was appraised by two reviewers independently using the AGREE‐GRS quality assessment instrument [49]. The final scores for each domain of the AGREE‐GRS tool were calculated based on the method recommended in the AGREE II User's Manual [50]. Scores of 33% and below for each domain were considered to be of low quality, scores between 34% and 66% were considered to be of moderate quality, and scores of 67% and more were considered to be of high quality. Guidance resources were not excluded based on the quality scores. The scores were used to compare the variation in methodological quality across guidance resources, which were then categorised as low, moderate, and high quality based on the AGREE‐GRS scores. This was used to inform judgement on the level of confidence in the evidence contributing to the final recommendation statements using principles from the GRADE‐CERQual (Grading of Recommendations Assessment, Development and Evaluation–Confidence in Evidence from Reviews of Qualitative research) approach [51, 52, 53, 54, 55, 56].

### Data extraction and data items

Using best practice guidance from the JBI Manual for Evidence Synthesis [57], a pre‐piloted digital data extraction form was customised in Excel, which was adapted from the JBI ‘text and opinion data extraction tool’ [58] to incorporate data fields specific to systematic reviews of textual opinion‐based evidence. The following specific data items were extracted from each resource:

- population – target audience for which guidance is developed;
- context within trial – areas of pathology input within clinical trial;
- phenomena of interest – variables associated with pathology‐specific trial guidance (guidance development methodology, organisational and geographical representation contributing to guidance development, reference to clinical research regulatory authority, clinical specialty focus, presence of a pathology‐specific trial protocol checklist);
- verbatim extracts of guidance statements; and
- interpretation of guidance statements; classifying guidance statements as ‘explicit’ or ‘implicit’ (explicit – ‘distinctly expressing all that is meant; leaving nothing merely implied or suggested’; implicit – ‘suggested or understood but not directly expressed’) [59].

Data from each selected resource were independently extracted by two reviewers onto the data extraction form, followed by checks for consistency. Any discrepancies were first discussed between the two reviewers, and any disagreements were resolved by a third reviewer.

### Data synthesis and analysis

We used the best fit framework synthesis method [60, 61, 62], which incorporates all elements of the JBI meta‐aggregation approach [63, 64], for synthesis and analysis of descriptive qualitative data. The best fit framework synthesis method incorporates techniques from both the framework synthesis and thematic analysis methods. We followed the five stages of this method, which involved ‘familiarisation’ with the literature to select an appropriate *a priori* framework (the SPIRIT 2013 Statement [6] was used as the *a priori* framework). This framework was applied to the ‘indexing’, ‘charting’, and ‘mapping and interpretation’ of extracted guidance statements in the data extraction, synthesis, and analysis stages of the review, respectively.

## Results

### Selection of guidance resources

**Figure 1:**
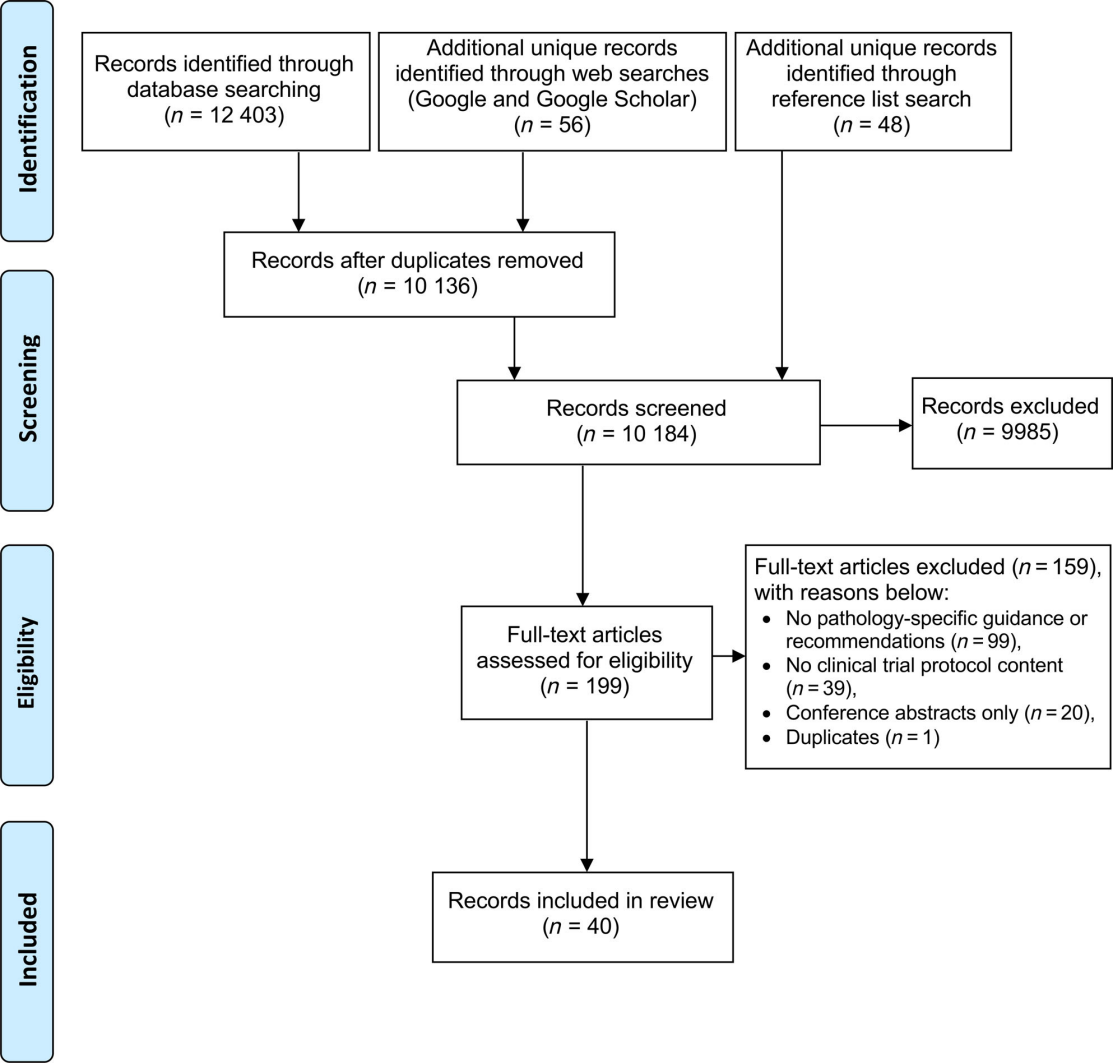
PRISMA flow diagram of selection process of guidance resources.

The database searches identified 12 403 records: 2706 from MEDLINE (Ovid), 5264 from EMBASE (Ovid), and 4433 from Cochrane Library. The web searches identified another 56 unique records. The supplementary search of reference lists yielded an additional 48 unique records, of which 12 full‐text articles were eligible for inclusion. No grey literature was identified. The titles and abstracts of 10 184 records were screened after de‐duplication. A total of 9985 records were excluded by screening titles and abstracts (*κ* = 0.76, substantial agreement). From the 199 full‐text articles retrieved and assessed for eligibility, 40 articles were included in the review (*κ* = 0.65, substantial agreement). There were no foreign language articles. The other 159 full‐text articles were excluded with reasons stated in Figure 1.

### Characteristics of guidance resources

Of the 40 guidance resources included [7, 9, 11, 12, 13, 14, 15, 16, 17, 18, 19, 20, 21, 22, 23, 24, 25, 26, 27, 28, 29, 30, 31, 32, 34, 35, 36, 37, 38, 39, 40, 41, 42, 65, 66, 67, 68, 69, 70, 71], around half (*n* = 21, 53%) offered recommendations pertaining to all aspects of pathology input within clinical trials (patient selection, risk stratification, and outcome assessment) [7, 9, 11, 13, 17, 20, 26, 27, 28, 29, 30, 35, 36, 38, 41, 42, 65, 67, 68, 69, 70]. Seven (17%) of the included guidance resources contained explicit recommendations [7, 18, 31, 39, 41, 66, 71], 24 (60%) contained implicit recommendations [9, 11, 12, 13, 14, 15, 16, 19, 22, 23, 24, 25, 27, 28, 29, 30, 34, 35, 36, 37, 42, 67, 69, 70], and the remaining 9 (23%) offered a combination of explicit and implicit recommendations [17, 20, 21, 26, 32, 38, 40, 65, 68]. Only four guidance resources had a pathology‐specific trial protocol checklist [17, 18, 32, 34]. A third of the guidance resources (*n* = 13) referenced one or more clinical research regulatory authority [9, 11, 18, 22, 25, 26, 32, 38, 40, 65, 67, 68, 69]. Detailed characteristics of individual guidance resources are provided in supplementary material, Table S1.

### Quality assessment

**Figure 2:**
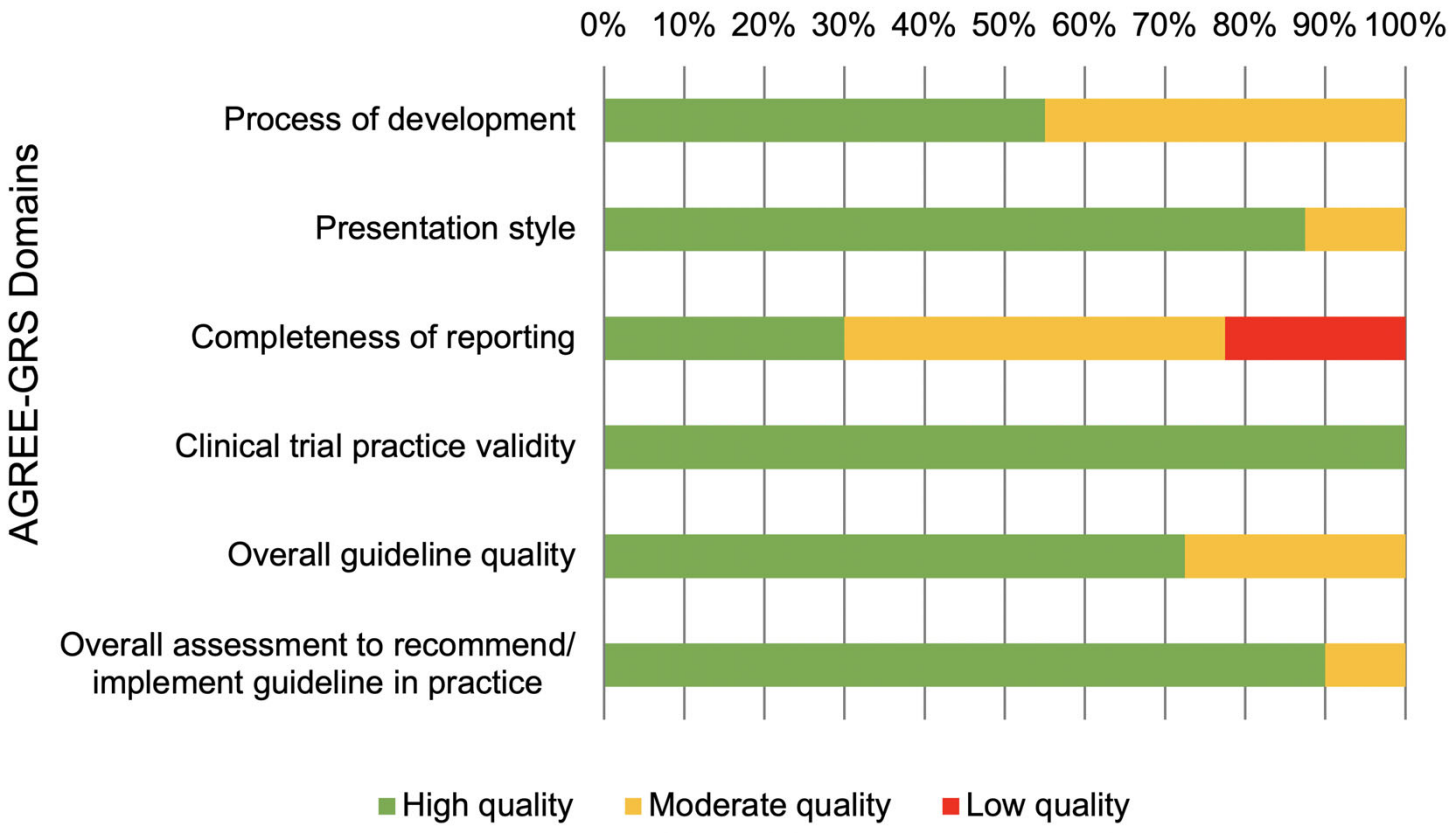
Proportion of guidance resources showing low, moderate, and high quality across AGREE‐GRS domains.

The quality scores for each AGREE‐GRS domain (out of 100%) for individual guidance resources are available in supplementary material, Table S2. All guidance resources were considered to be highly relevant and applicable to clinical trial practice (Figure 2). The majority of the guidance resources was assessed to have high quality (AGREE‐GRS score of >67%). However, the quality was considered to be lower (AGREE‐GRS score of <33%) in the AGREE‐GRS domain of ‘completeness of reporting’, subsequently mapping to the GRADE‐CERQual domain of ‘adequacy of data’.

### Heterogeneity across guidance resources

Sources of heterogeneity that could contribute to bias across guidance resources are listed in Table 1. The majority (58%) of the included guidance resources was discussion papers (including perspectives or opinion papers), while the remainder consisted of a mixture of literature review, primary research papers, letters to editors, regulatory authority guidance documents, and methodology papers. The majority of the resources (73%) were published in the last decade, whereas only two (5%) were published between 1996 and 2005; the remaining were published between 2006 and 2010. In terms of the guidance development process, nearly half (45%) of the resources had employed a rigorous development methodology involving a formal working group at a national or international level, while 15 (38%) did not report a formal methodology. The majority of these 15 that did not report a formal methodology (*n* = 11) was based on the opinions, perspectives, and experiences of a selected group of authors. Nearly half of the guidance resources (45%) were recommendations developed by the United States and/or Canada, whereas only a quarter were developed by the United Kingdom (*n* = 5) and other countries within Europe (*n* = 5). Only 10 resources (25%) included recommendations developed from an international representation of experts.

**Table 1:** Heterogeneity across guidance resources.

Sources of heterogeneity		Number of resources (%)
Type of publication	Discussion paper	23 (57.5)
	Literature review	4 (10.0)
	Research paper (primary studies)	3 (7.5)
	Short communication (letter)	2 (5.0)
	Regulatory authority guidance document	4 (10.0)
	Methods paper	3 (7.5)
	Book chapter	1 (2.5)
Year of publication	1996–2000	1 (2.5)
	2001–2005	1 (2.5)
	2006–2010	9 (22.5)
	2011–2015	16 (40.0)
	2016–2020	13 (32.5)
Methodology used in guidance development	Formal consensus process involving international stakeholders	9 (22.5)
	National Task Force consensus or formal working group consensus	9 (22.5)
	Review of literature	4 (10.0)
	Conclusions from primary studies	3 (7.5)
	Guideline development methodology unreported	15 (37.5)
	Opinions or perspectives of a single author or few authors	11 (27.5)
	Regulatory agency guidance document	3 (7.5)
	Book chapter	1 (2.5)
Geographical representation of experts involved in guidance development	USA only	15 (37.5)
	North America only	3 (7.5)
	UK only	5 (12.5)
	The Netherlands only	1 (2.5)
	Europe only	4 (10.0)
	North America and Europe	2 (5.0)
	International	10 (25.0)

### Results of individual guidance resources

The verbatim extracts of recommendation statements from each individual guidance resource, mapped onto the *a priori* framework (the SPIRIT 2013 Statement) [6], followed by its associated interpretation and ‘charting’ are available on the Open Science Framework online repository [72]. During the charting stage, the recommendation statements from the individual guidance resources were coded into themes that best describe the nature and type of recommendations offering best practice guidance at various stages of the clinical trial process. The charting process revealed that the themes could be broadly categorised according to stages of the clinical trial process, namely, pre‐analytical, analytical, and post‐analytical phases, as well as across all phases of the trial. Table 2 shows the frequency and distribution of resources offering guidance for each identified theme.

**Table 2:** Results of individual guidance resources showing types of recommendations for pathology input in clinical trials.

Recommendation category	Specific recommendation	Number of resources	%	References
Pre‐analytical phase	Personnel			
	Accreditation and training of pathologists	8	20	[9, 11, 13, 25, 38, 65, 68, 69]
	Accreditation and training of laboratory staff	4	10	[9, 13, 65, 68]
	Statistical and technical laboratory expertise for pathology‐based parameters	8	20	[11, 16, 17, 18, 26, 32, 42, 69]
	General laboratory systems and facilities			
	Laboratory accreditation	10	25	[7, 11, 13, 18, 25, 28, 38, 65, 68, 69]
	Assay validation and performance testing	4	10	[13, 17, 18, 25]
	Standardisation of laboratory processes (including standard operating procedures)	4	10	[11, 13, 65, 68]
	Rationale for pathology‐specific criteria			
	Use of biospecimens, specific biomarker, or associated analytical assay	8	20	[17, 18, 22, 24, 25, 32, 67, 71]
	Inclusion criteria or risk stratification	3	7.5	[22, 25, 32]
	Outcome measurement	3	7.5	[22, 32, 41]
	Biospecimens			
	Standardisation of sample collection and handling procedures	7	17.5	[7, 17, 19, 25, 28, 67, 70]
	Sample storage conditions	5	12.5	[19, 28, 67, 68, 70]
	Sample transport conditions across sites	7	17.5	[17, 19, 25, 28, 67, 68, 70]
	Biobank facilities	4	10	[19, 28, 67, 70]
	Disease‐specific pre‐analytical sampling and processing methods (see supplementary material, Table S3)	4	10	[20, 21, 34, 36]
	Multidisciplinary collaboration among all parties involved in trial	10	25	[7, 9, 11, 17, 18, 24, 25, 26, 65, 67]
	Funding of materials, laboratory staff, and pathologists	4	10	[11, 18, 32, 38]
Analytical phase	Microscopic assessment methods			
	Use of artificial intelligence for microscopic analysis	2	5	[29, 38]
	Central pathology review	3	7.5	[11, 35, 66]
	Histopathology reporting	2	5	[7, 68]
	Disease‐specific analytical methods (see supplementary material, Table S3)	8	20	[12, 14, 15, 23, 31, 34, 37, 40]
	Auditing and data validation	8	20	[11, 13, 18, 30, 35, 38, 66, 68]
Post‐analytical phase	Dissemination of results			
	Data sharing	3	7.5	[19, 28, 70]
	Transparent reporting	3	7.5	[27, 28, 70]
Across all trial phases	Data monitoring and validation	5	12.5	[18, 30, 42, 65, 68]
	Informed consent materials and supporting documentation to be given to participants	8	20	[11, 17, 18, 24, 39, 41, 67, 69]
	Confidentiality and data protection	5	12.5	[18, 25, 41, 67, 68]
	Ethics surrounding biospecimens collection, handling, storage, and transport	4	10	[24, 39, 41, 67]
	Ethics surrounding genetic testing and data sharing	2	5	[25, 67]

The recommendation statements categorised according to recurring themes form the basis of synthesised recommendations. Other guidance resources offering standalone recommendations do not contribute to any recurring themes and are therefore not generalisable enough for synthesis; these are discussed below.

Five of the guidance resources contain recommendations specific only to certain types of clinical trials. These include best practice guidance for specific laboratory techniques such as tissue microarray construction and evaluation [42], consideration of laboratory technical parameters in omics‐based clinical trials [26], and the technical aspects of biomarker development [16] and biomarker integration [18, 25] in early‐phase clinical trials.

Eleven of the guidance resources offer recommendations focused on the pre‐analytical and analytical phases of clinical trials specific to a clinical specialty, such as breast cancer [12, 20, 21, 31, 40], non‐small cell lung cancer [34], paediatric neuroblastoma [37], paediatric rheumatology [36], prostate cancer [14], and lymphoma [15, 23]. Details of these disease‐specific recommendations are shown in supplementary material, Table S3.

The guidance documents from the European Medicines Agency [65] and the Medicine and Healthcare products Regulatory Agency [68] stipulate the minimum regulatory standards to which all laboratory supporting clinical trial work should adhere. As meeting these standards is a legal requirement, these two resources have not been included in recommendation synthesis.

### Synthesis of recommendations

Best practice recommendation statements that contributed to the recurring themes were gathered and synthesised from 22 of the guidance resources [7, 9, 11, 13, 17, 19, 22, 24, 27, 28, 29, 30, 32, 35, 38, 39, 41, 66, 67, 69, 70, 71] using the JBI meta‐aggregation approach (see supplementary material, Table S4). The GRADE‐CERQual evidence profile (see supplementary material, Table S5) shows the CERQual assessment details, with reasons for reaching the judgements, for each of the four GRADE‐CERQual components. The synthesised pathology‐specific recommendations at different stages of the clinical trial process, along with the overall confidence in each synthesised statement, are as follows:

#### Pre‐analytical phase

*Recommendation 1* (*R1*): The responsibilities and level of involvement in clinical trial work should be agreed upon among all multidisciplinary collaborators and formally documented prior to trial opening. Input from pathologists and other relevant personnel with technical laboratory and statistical expertise and experience should be sought during the development of trial protocol, trial design, and implementation, in particular for justifying the use of biospecimens and/or a specific biomarker and associated analytical assays in the clinical trial, as well as reaching a consensus on pre‐specified definitions of pathology‐related parameters when interpreting findings for trial inclusion or risk stratification. (Moderate confidence).

*Recommendation 2* (*R2*): All personnel undertaking any aspect of clinical trial work should have proper accreditation and sufficient funding and training corresponding to their involvement and role in the clinical trial. (High confidence).

*Recommendation 3* (*R3*): The laboratory site selected to carry out trial work should have:

- appropriate accreditation, with practices of laboratory management and operations complying with standards of regular external quality assurance schemes;
- capacity to adhere to trial‐specific standard operating procedures in the testing platforms and preparation and storage of sample; and
- suitable facilities required for the trial (e.g. accredited digital pathology platforms). (Moderate confidence).

#### Analytical phase

*Recommendation 4* (*R4*): Plans to ensure the completeness and accuracy of pathology‐related data sets in clinical trials should be clearly documented and should address the following:

- a system for prospective rapid real‐time central pathology review or double reporting with consensus to achieve uniformity in diagnosis;
- use of standardised digital pathology platforms where appropriate;
- data quality review by a trained pathology quality manager or review committee to ensure adherence to standardised pathology examination and interpretation procedures; and
- regular analytical audits of internal testing platforms and assay performance testing and validation of inter‐pathologist reproducibility and inter‐laboratory repeatability analysis. (High confidence).

#### Post‐analytical phase

*Recommendation 5* (*R5*): The pathology‐relevant aspects of clinical trials should be transparently reported according to the BRISQ (Biospecimen Reporting for Improved Study Quality) checklist and REMARK (REporting recommendations for tumour MARKer prognostic studies) criteria, where appropriate, to include specific details relevant to biospecimen procurement, type, anatomical site, and associated patients' clinical details, as well as protocols for tissue preparation, preservation, and biomarker staining parameters. These standard operating procedures for the management of biospecimens during the clinical trial should be registered on a publicly accessible database, with digital location cited in the research publication. (Low confidence).

#### Across all trial phases

*Recommendation 6* (*R6*): Laboratories and biorepositories should have policies and procedures with secure informatics systems in place to minimise risks of harm to participants and to protect the confidentiality and data of participants, including anonymising collected biospecimens, tracking the movement of biospecimens within and across sites, and ensuring consent for biospecimen retention is valid prior to storage. (Moderate confidence).

*Recommendation 7* (*R7*): Patient information sheets and consent forms should be reviewed by pathologists and should include information pertaining to the rationale of the use of biospecimen within the context of the clinical trial; the risks and benefits involved; how data will be analysed, stored, transferred between institutions, and shared with their healthcare providers; the details of biospecimen collection (type, frequency, volume, or size of sample); and specimen retention policies for future research. In studies involving genomics or genetics, participants should specifically be counselled on the implications of a positive result on themselves and their relatives. (Moderate confidence).

## Discussion

### Applicability of synthesised findings to clinical trial practice

The findings of this review yielded seven synthesised recommendation statements, covering the pre‐analytical, analytical, and post‐analytical phases of clinical trials, as well as guidance pertaining to biospecimen ethics and informed consent documentation, which spans across all phases of the clinical trial process. The GRADE‐CERQual Summary of Qualitative Findings (see supplementary material, Table S6) shows a summary of the synthesised recommendations and their respective overall quality. It also shows the applicability of each of the seven synthesised recommendation statements to particular aspects of the clinical trial process and the relevance of each recommendation to different groups of key clinical trial stakeholders, as well as the implications of each recommendation on potentially changing clinical trial practice in trial protocol development and trial reporting, within the context of the SPIRIT Statement [6] and CONSORT Statement [73] checklist items.

### Strengths and limitations

This systematic review has been transparently reported according to the PRISMA 2009 Statement. Selection bias of resources was minimised by having no restrictions placed on language and publication type and by two reviewers independently selecting the studies for inclusion. Publication bias was mitigated by searching several databases and the web. Furthermore, this systematic review has been conducted in accordance with the international best practice guidance provided by the JBI Manual for Evidence Synthesis [57] for this type of reviews of text and opinion, except for the use of alternative assessment tools for appraising the quality of individual studies (AGREE‐GRS used instead of ‘JBI‐Qualitative Critical Appraisal Checklist’) and in establishing confidence in the synthesised evidence (GRADE‐CERQual used instead of the ConQual approach). The AGREE‐GRS tool was used because its checklist questions are more suitable and appropriate for use to evaluate the quality of the nature of content (recommendations and guidelines) presented in the papers within this systematic review compared to that of the JBI‐Qualitative Critical Appraisal Checklist [57]. The GRADE‐CERQual approach to establishing confidence was subsequently adopted as the domains of AGREE‐GRS map better onto the GRADE‐CERQual assessment domains than the ConQual assessment domains. During the data synthesis process of this systematic review, the formulation of recommendation statements and the quality assessment of cumulative evidence using the CERQual approach were determined through the consensus of three reviewers.

Individual guidance resources were found to be heterogeneous. The majority of publications were in the last decade, suggesting relevance to current pathology‐related practices within clinical trials, such as the use of digital pathology in data quality assurance and central pathology review. However, more than half of the resources included were discussion papers in the form of opinion papers and perspective papers, which is the main limitation to this review in terms of status on the evidence hierarchy. In fact, most of the synthesised recommendations with overall confidence downgraded to ‘moderate’ were due to ‘moderate methodological limitations’ on the CERQual assessment as a result of incomplete documentation of the guideline development process within the working groups that published the perspective papers. Furthermore, the preponderance of publications with organisational representation in the Western countries, particularly in the North American countries, may introduce bias and result in synthesised recommendations that are not generalisable to trials conducted in other geographical locations.

### Suggestions for future guidance developments

Evidence from this systematic review suggests that the current literature contains recommendations addressing most aspects of pathology input into clinical trials. However, it has not kept up with domains within pathology that have evolved with recent rapid advancement in technologies used for developing personalised medicine, which have revolutionised conventional diagnostic pathology.

There is scope for future developments in pathology‐specific guidance into clinical trials in terms of transparent reporting of technology‐dependent pathology parameters, particularly pertaining to the use of standardised digital platforms for histopathological assessment, as well as techniques for biomarker analysis in the molecular classification of diseases. Recommendations for genomic profiling methods used in molecular diagnostics, which have clinical implications for treatment, disease monitoring, and prognostication, are also currently lacking. These gaps in the literature can be effectively addressed by appropriate guideline development initiatives [74].

## Conclusions

This systematic review to synthesise pathology‐specific recommendations within clinical trials is the first of its kind. The findings will inform an international effort to develop a pathology extension of the SPIRIT Statement [6], called SPIRIT‐Path [74], using the Delphi consensus method involving stakeholders from diverse backgrounds. Implementation of pathology‐specific best practice recommendations into clinical trial protocols will enhance the methodological and scientific rigour of trial delivery, improving the reliability of evidence, and translating into rational healthcare improvements for the benefit of patients worldwide.

## Author contributions statement

DO conceived the idea. All the authors contributed to the development of the protocol. KG provided methodological advice. SJL, TJK and MR extracted, analysed, and synthesised the data. SJL and MR wrote the first draft of the manuscript, and all authors critically reviewed and approved the final version of the manuscript.

## Supporting information


**Table S1.** Characteristics of included guidance resources
**Table S2.** Quality assessment of individual guidance resources
**Table S3.** Clinical specialty‐specific recommendations for biospecimen collection, processing, and histopathological assessment in clinical trials
**Table S4.** Meta‐aggregative synthesis flowchart of pathology‐specific recommendations according to different stages of the clinical trial process
**Table S5.** GRADE‐CERQual evidence profile
**Table S6.** GRADE‐CERQual summary of qualitative findings
