# Supplementary material for: Recommendations for cellular and molecular pathology input into clinical trials: a systematic review and meta‐aggregation
Source: J Pathol Clin Res. 2021 Feb 26;7(3):191–202. doi: 10.1002/cjp2.199 (PMC8073003; doi:10.1002/cjp2.199)
Supplement: Supplementary file 1 — Table S1. Characteristics of included guidance resources Table S2. Quality assessment of individual guidance resources Table S3. Clinical specialty‐specific recommendations for biospecimen collection, processing, and histopathological assessment in clinical trials Table S4. Meta‐aggregative synthesis flowchart of pathology‐specific recommendations according to different stages of the clinical trial process Table S5. GRADE‐CERQual evidence profile Table S6. GRADE‐CERQual summary of qualitative findings [file CJP2-7-191-s001.docx]

**Recommendations for cellular and molecular pathology input into clinical trials: a systematic review and meta-aggregation**

SJ Lim *et al*, *J Pathol Clin Res*, DOI 10.1002/cjp2.199

**Table S1.** Characteristics of included guidance resources.

| **Authors, Year (Source)** | **Publication type** | **Aim** | **Target population for which guidance is developed** | **Characteristics of pathology-specific guidance** | | | | | | |  |
| --- | --- | --- | --- | --- | --- | --- | --- | --- | --- | --- | --- |
|  |  |  |  | Guidance development methodology | Organisational and geographical representation in guidance development process (country/ region) | Regulatory authority referenced | Clinical area  in which guidance is focused | Pathology-specific trial protocol checklist provided | Recommendation type  *(Implicit/ Explicit)* | **Area(s) of pathology input within clinical trial** | |
| Bossuyt et al., 2015 (Annals of Oncology)  (1) | Methods paper | To recommend practical methods for standardized evaluation of the post-neoadjuvant systemic therapy breast cancer specimens in clinical trials that promote accurate and reliable designation of pathologic complete response and meaningful characterization of residual disease | All collaborators involved in breast cancer clinical trials | • Review of trial SOPs to formulate recommendations • Consensus opinion of international group of experts sought where evidence was lacking | BIG-NABCG collaboration (International) | None | Breast cancer | No | Explicit | Outcome assessment | |
| Dancey et al., 2010 (Clinical Cancer Research) (2) | Discussion paper | To give an overview of standard definitions and categories of biomarkers, and provide recommendations to sponsors and investigators for biomarker incorporation into early clinical trials | Sponsors and investigators of biomarker studies in early clinical trials | • Review of past and current biomarker studies, peer-reviewed literature and guidance documents from NCI and FDA • Survey of early clinical trial investigators' perspectives • Consensus of Task Force members | Biomarker Task Force of the NCI IDSC (North America) | FDA | General | Yes | Implicit & Explicit | Selection; Stratification | |
| Eberhard et al., 2008 (Journal of Clinical Oncology) (3) | Discussion paper | To present recommendations for the standardization of EGFR molecular assays for its use and interpretation in the clinical trial setting, including guidelines for tissue storage, handling, and processing | Oncologists, pathologists, scientists with expertise in molecular testing, and biostatisticians involved in NSCLC clinical trials | • Recommendations informed by published and unpublished data from primary studies • Formal working group expert consensus (multidisciplinary group of 20 individuals) following meeting discussions | Molecular Assays in NSCLC Working Group (representatives from North America, EU, UK) | None | Lung cancer | Yes | Implicit | Outcome assessment | |
| European Medicines Agency, 2012 (Self-published web-based document)(4) | Guidance document | To provide laboratories that perform analysis or evaluation of human samples collected as part of a clinical trial, with information that will help them develop and maintain quality systems which will comply with relevant European Union Directives, national regulations and associated guidance documents | Contract research organisations, sponsors laboratories and non-commercial laboratories | Formal working group expert consensus with public consultation | EMA GCP Inspectors Working Group (EU) | EMA | General | No | Implicit & Explicit | Selection; Stratification; Outcome assessment | |
| Hall et al., 2011 (Biopreservation and Biobanking) (5) | Discussion paper | To present considerations required for standardizing collection of human biological materials in clinical trials and the approaches taken by EORTC to integrate such collections in clinical trial protocols | Research investigators, pathologists, biobankers, biostatisticians, basic scientists, physicians | • Opinion-based recommendations (multidisciplinary group of 6 experts of a single organisation) • Informal discussions and input from members of EORTC Translational Research Advisory Committee, Pharmacology and Molecular Mechanisms Group, PathoBiology Group, Translational Research Unit and EORTC headquarters staff | EORTC (EU) | None | Oncology | Yes | Implicit & Explicit | Selection; Stratification; Outcome assessment | |
| Hall et al., 2014 (The Lancet Oncology) (6) | Discussion paper | To identify challenges associated with biomarker integration in phase II and III clinical trials of solid and haematological cancers, and to present a risk-assessment framework and checklist of recommendations to help principal investigators with protocol development for biomarker-driven trials | Principal investigators and clinical trial stakeholders (regulators, public authorities, patients) | Opinion-based recommendations  (5 experts from 3 clinical trial organisations) | NCI CDP, EORTC PathoBiology Group, NCRI Biomarkers and Imaging Clinical Studies group (USA, EU, UK) | FDA, EMA, MHRA | General | Yes | Explicit | Selection; Stratification | |
| LaBaer, 2012 (Journal of Proteome Research) (7) | Discussion paper | To highlight the major challenges that hinder the use of clinical specimens for translational research, and to propose solutions to overcome these challenges | Researchers, biobankers, pathologists, assay developers and other personnel involved in proteomics translational research | Personal opinion (single author) | Biodesign Institute (USA) | None | General | No | Implicit | Selection; Stratification | |
| LaBaer et al., 2018 (Nature Methods) (8) | Short communication (Letter) | To highlight the need for transparency in biospecimen procurement methods and provide suggestions for completeness of reporting in biospecimen processing to improve research reproducibility | Biorepositories, journals, funding agencies, researchers | Opinion-based suggestions (3 authors) | Biodesign Institute and Global Biological Standards Institute (USA) | None | General | No | Implicit | Selection; Stratification; Outcome assessment | |
| Leyland-Jones et al., 2008 (Journal of Clinical Oncology) (9) | Discussion paper | To present consensus guidelines for procedures of collection, processing, handling and shipment of biospecimens collected from the BIG-NABCG breast cancer clinical trials | Tissue procurement sites | • Evaluation of previous biobanking SOPs • Questionnaire to international trial groups • Expert consensus from 3 working groups | BIG, North American Cooperative Groups, NCI CTEP, NCI CDP, NCI GBC (International) | None | Breast cancer | No | Implicit & Explicit | Selection; Stratification; Outcome assessment | |
| Loi et al., 2011 (The Lancet Oncology) (10) | Discussion paper | To outline proposals for uniform and standardised collection of high-quality biospecimens obtained in neoadjuvant breast cancer trials undertaken by the BIG-NABCG collaboration in order to enhance and allow integration of results obtained from neoadjuvant trials done by several groups | Clinical trial groups involved in neoadjuvant breast cancer trials | Expert consensus from international multidisciplinary working group | BIG, NABCG (International) | None | Breast cancer | No | Implicit & Explicit | Selection; Outcome assessment | |
| LoRusso et al., 2010 (Clinical Cancer Research) (11) | Discussion paper | To present consensus recommendations for the optimal planning, design and conduct of phase I studies, with focus on efficient trial designs, drug combinations and appropriate statistical correlative endpoints | Scientific, medical and statistical communities involved in phase I clinical trials of new therapeutics | Consensus of Task Force members following workshop discussion | Clinical Trial Design Taskforce of the NCI IDSC (North America) | FDA | General | No | Implicit | Outcome assessment | |
| Maddocks et al., 2017 (Journal of the National Cancer Institute)  (12) | Literature review | To highlight the current landscape of clinical trials in follicular lymphoma, provide recommendations for future directions of follicular lymphoma clinical trials and suggestions for inclusion of biomarkers, novel therapeutic agents and assessment of trial endpoints in future trials | Collaborators involved in follicular lymphoma clinical trials | Overview of literature from recent and ongoing trials in follicular lymphoma | NCI Lymphoma Clinical Trials Planning Meeting Follicular Subcommittee (USA) | None | Lymphoma | No | Implicit | Stratification; Outcome assessment | |
| Makhlouf et al., 2019 (American Journal of Clinical Pathology) (13) | Discussion paper | To identify institutional barriers and provide strategic recommendations to facilitate the submission of diagnostic tissue blocks for clinical trials | Clinical trialists and research staff, pathology departments, clinical research coordinators, surgeons, oncologists, pathologists, biobankers, regulatory agencies, patient advocates | Formal expert consensus from a multidisciplinary group following workshop discussion | NCI (USA) | None | Oncology | No | Implicit | Selection | |
| Marton & Weiner, 2013 (BioMed Research International) (14) | Discussion paper | To review common issues encountered when implementing predictive biomarkers into early phase clinical studies, with focus on key practical issues that should be considered by clinical teams when planning to use a biomarker to balance arms of a study or determine eligibility for a clinical study | Drug development teams comprising of clinicians, discovery scientists, biomarker experts, regulatory personnel, statisticians, assay developers | Opinion-based suggestions (2 authors) | Merck Research Laboratories (USA) | FDA | General | No | Implicit | Selection; Stratification; Outcome assessment | |
| McShane & Hayes, 2012 (Journal of Clinical Oncology) (15) | Literature review | To review the current state of efforts to enhance the quality and transparency of reporting of tumor marker studies | Researchers, study funders, regulatory agencies, payers, patients, editors of journals that publish oncology research | Overview of literature on reporting recommendations for studies using biospecimen and tumour biomarkers | NCI CDP and University of Michigan Comprehensive Cancer Centre (USA) | None | Oncology | No | Implicit | Selection; Stratification; Outcome assessment | |
| McShane et al., 2013 (Nature) (16) | Discussion paper | To present a checklist of criteria that should be addressed to determine the readiness of omics-based tests for guiding patient care in clinical trials | Clinical trialists, pathologists, laboratory staff, bioinformaticians, trial data managers, statisticians, funding bodies, journal reviewers and editors | Formal working group expert consensus following workshop discussion (multidisciplinary group) | Institute of Medicine and NCI (USA) | FDA | General | No | Implicit & Explicit | Selection; Stratification; Outcome assessment | |
| Medicine and Healthcare products Regulatory Agency, 2009 (Self-published web-based document) (17) | Guidance document | To provide organisations that participate in any aspect of a human clinical trial with information that will enable them to develop and maintain quality systems which will comply with the Medicines for Human Use (Clinical Trials) Regulations | All persons and facilities that perform clinical trial work | Regulatory standards incorporating EU statutory instruments | MHRA (UK) | MHRA | General | No | Implicit & Explicit | Selection; Stratification; Outcome assessment | |
| Moore et al., 2011 (Cancer Cytopathology) (18) | Discussion paper | To provide recommendations for the reporting of data elements for studies in which human biospecimens are used, such as clinical trials, translational science, biomarker discovery, technology development, diagnostic-assay and therapeutics development | Researchers, regulatory agencies | • Expert consensus from an international multidisciplinary working group after an initial workshop discussion followed by monthly teleconferences • Literature from peer-reviewed articles in NCI Biospecimen Research Database | BRISQ Committee (International) | None | General | No | Implicit | Selection; Stratification; Outcome assessment | |
| Moorthy, 2018 (The Lancet Oncology) (19) | Short communication (Letter) | To identify the oversight in the assignment of treatment groups in melanoma-related clinical trial protocols that could have affected the trials' conclusions | Collaborators involved in targeted therapy clinical trials (clinical investigators, trained laboratory personnel, pathologists, assay developers), regulatory organisations | Personal opinion (single author) | MultiGEN Diagnostics (USA) | None | Oncology | No | Explicit | Stratification | |
| Mroz et al., 2013 (Archives of Pathology & Laboratory Medicine) (20) | Literature review | To review the current experience with central pathology review in clinical trials, summarize current developments in virtual microscopy and discuss advantages and disadvantages of virtual microscopy in central pathology review | Individuals who are part of oncology cooperative groups undertaking clinical trial work | Non-systematic review of PubMed-indexed publications | Northwestern University, Harvard Medical School, University of Pittsburgh (USA) | None | General | No | Implicit | Selection; Stratification; Outcome assessment | |
| Nagtegaal et al., 2000 (Journal of Clinical Oncology) (21) | Research paper | To examine the completeness and accuracy of data obtained from pathology case report forms used in trials compared to hospital pathology reports | Review pathologists, trial data managers, pathology quality managers | Conclusions from primary study, based on data from 300 patients with rectal cancer from a single large multicentre trial | Leiden University Medical Centre, University Medical Centre St Radboud, the Pathology Review Committee (The Netherlands) | None | General | No | Implicit | Selection; Stratification; Outcome assessment | |
| Nagtegaal et al., 2014 (Journal of Pathology) (22) | Discussion paper | To highlight the essential roles of pathologists in the design and execution of oncology trials, particularly in the assessment of eligibility and stratification, and the evaluation of response to therapy | All collaborators involved in prevention and screening trials, surgical trials, radiotherapy, immune approaches or viral therapy trials | • Opinion-based suggestions (4 authors) • Data from current trials cited as examples | St James's University Hospital, Radboud University Nijmegen Medical Centre (UK, The Netherlands) | None | Oncology | No | Explicit | Selection; Stratification; Outcome assessment | |
| National Cancer Institute, 2016 (Self-published web-based document) (23) | Guidance document | To identify guiding principles that define current evidence-based best practices for biospecimen resources, promote biospecimen and data quality, and support adherence to ethical and legal requirements | Clinical investigators, biospecimen resource personnel, biospecimen custodians, pathologists, clinical research coordinators and study nurses, research assistants, laboratory technicians, bioinformaticians, statisticians | • Synthesis of regulatory standards documents and legal policies with results from biospecimen research initiatives • Input from stakeholders in cancer research community (research participants, patient advocates, researchers, clinicians, biospecimen resource personnel) | NCI (USA) | FDA | Oncology | No | Implicit | Selection; Stratification; Outcome assessment | |
| Park et al., 2017 (Journal of Clinical Oncology) (24) | Discussion paper | To develop and implement an updated revised international consensus response criteria for newly diagnosed, refractory or recurrent neuroblastoma | Clinical trialists, trial pathologists, trial radiologists involved in paediatric neuroblastoma trials | • Evaluation of published trial data by a multidisciplinary group of 52 investigators from 13 countries • Expert consensus reached following monthly teleconferences and through review by working group leadership and the NCI Clinical Trials Planning Meeting leadership council | NCI-appointed executive planning committee (International representatives) | None | Paediatric oncology | No | Implicit | Outcome assessment | |
| Pell et al., 2019 (The Journal of Pathology: Clinical Research) (25) | Discussion paper | To provide an overview of the utility of digital pathology and image analysis technologies in clinical trials and discuss potential applications, challenges and limitations | Digital pathology community, clinical trial regulators and industry, software engineers, information network specialists | Formal working group expert consensus following workshop discussion (multidisciplinary group) | NCRI CM-Path quality assurance working group (UK) | FDA, MHRA | General | No | Implicit & Explicit | Selection; Stratification; Outcome assessment | |
| Peppercorn et al., 2010 (Journal of Clinical Oncology) (26) | Discussion paper | To review the context of research biopsies in oncology clinical trials, present and evaluate ethical concerns based on authors' experiences, and propose a framework for ethical conduct of mandatory biopsies within clinical trials | Clinical investigators involved in oncology trials, ethics committee | Opinion-based suggestions and perspectives of authors' experiences | Cancer and Leukaemia Group B Ethics Committee (USA) | None | Oncology | No | Explicit | Selection | |
| Pollack et al., 2003 (Neuro-Oncology) (27) | Research paper | To evaluate the effects of discordant diagnoses between single-expert review and central pathology review on the outcome results (therapeutic efficacy and prognostic correlates) of a randomized paediatric high-grade gliomas trial | Clinical trialists, trial pathologists | Conclusions from primary study, based on data from 172 children with high-grade glioma from a single multicentre trial | Children's Cancer Group (USA) | None | Paediatric oncology | No | Explicit | Selection | |
| Provenzano et al., 2015 (Modern Pathology) (28) | Methods paper | To develop practical recommendations for the pathologic assessment of residual disease in neoadjuvant breast cancer clinical trials and the information expected from pathology reports | Breast cancer trial pathologists | • Evaluation of trial SOPs and site-specific SOPs for evidence of variability in tissue handling and histological assessment • Expert consensus from an international multidisciplinary working group | Residual Disease Characterization Working Group of the BIG-NABCG collaboration (International) | FDA | Breast cancer | No | Implicit & Explicit | Outcome assessment | |
| Rees et al., 2019 (The Journal of Pathology: Clinical Research) (29) | Discussion paper | To recommend the optimal training and accreditation for pathologists undertaking clinical trial activities in order to ensure high quality pathology in clinical trials | Trial pathologists | Formal working group consensus of pathologists following workshop discussion | NCRI CM-Path clinical trials working group (UK) | MHRA | General | No | Implicit | Selection; Stratification; Outcome assessment | |
| Ricci & Franc, 2008 (Pharmacogenomics and Personalized Medicine) (30) | Book chapter | To provide an overview of the study design elements on an operational level and the ethical and logistical considerations during the implementation of pharmacogenomic sample collection in clinical trials | All individuals involved in pharmacogenomics-related clinical trials | Overview of guidance and policies written by 2 pharmaceutical industry authors | Johnson and Johnson Pharmaceutical Research and Development (USA) | None | General | No | Explicit | Selection; Stratification; Outcome assessment | |
| Rimm et al., 2011 (Journal of Clinical Oncology) (31) | Discussion paper | To propose guidelines for high-quality tissue microarray construction representing multicentre clinical trial tissues, and discuss statistical issues regarding missing tissue blocks | All laboratory staff and pathologists involved in clinical trials | Opinion-based suggestions and perspectives of authors' experiences as array facility directors | Cancer and Leukaemia Group B Pathology Committee (USA) | None | General | No | Implicit | Selection; Stratification; Outcome assessment | |
| Robinson et al., 2019 (The Journal of Pathology: Clinical Research) (32) | Discussion paper | To provide guidance for quality assurance of pathology scoring and reporting in clinical trials | All laboratory staff and pathologists involved in clinical trials | Formal working group expert consensus following workshop presentation and discussion (multidisciplinary group) | NCRI CM-Path clinical trials working group (UK) | MHRA | General | No | Implicit | Selection; Stratification; Outcome assessment | |
| Salgado et al., 2015 (Annals of Oncology) (33) | Methods paper | To review current data on the clinical validity and utility of tumour-infiltrating lymphocytes (TILs) in breast cancer and to develop consensus recommendations for TILs evaluation in primary tumour specimens before therapy | Researchers involved in breast cancer studies | • Systematic literature review of PubMed-indexed studies conducted by a multidisciplinary team • Expert consensus following teleconferences and email correspondences (panel members have had experience in TIL assessment) | International TILs Working Group | None | Breast cancer | No | Implicit | Outcome assessment | |
| Sarzotti-Kelsoe et al., 2009 (PLoS Medicine)  (34) | Discussion paper | To propose a harmonized interpretation of the Good Clinical Laboratory Practice elements (training, auditing, assay validation, proficiency testing) for optimal management of clinical laboratory operations to successfully support phase I to III clinical trials | Clinical laboratories supporting clinical trial work, all regulatory agencies | Expert consensus from an international collaborative group following workshop discussion | Global HIV Vaccine Enterprise, International AIDS Vaccine Initiative, NIH Division of AIDS, Qualogy Ltd (International) | None | General | No | Implicit | Selection; Stratification; Outcome assessment | |
| Scher et al., 2016 (Journal of Clinical Oncology) (35) | Discussion paper | To update and revise recommendations for trial design and objectives in castration-resistant prostate cancer clinical trials | All individuals involved in prostate cancer clinical trials | Expert consensus from international working group comprising of more than 20 clinical and translational experts in prostate cancer | Prostate Cancer Clinical Trials Working Group 3 (International) | None | Prostate cancer | No | Implicit | Outcome assessment | |
| Spurgeon et al., 2017 (Journal of the National Cancer Institute) (36) | Literature review | To highlight the current landscape of clinical trials in mantle cell lymphoma, identify priorities and directions for future mantle cell lymphoma clinical trials, and provide suggestions for inclusion of biomarkers, novel therapeutic agents and assessment of trial endpoints in future trials | Collaborators involved in mantle cell lymphoma clinical trials | Overview of literature from recently reported and ongoing trials in mantle cell lymphoma | NCI Lymphoma Clinical Trials Planning Meeting Committee (USA) | None | Lymphoma | No | Implicit | Stratification; Outcome assessment | |
| Stewart & Kurzrock, 2013 (BMC cancer) (37) | Discussion paper | To discuss the importance of early identification and development of predictive biomarkers in the course of clinical drug development and the appropriate methods of evaluating biomarker performance, and to suggest alternative phase II trial designs with new approach to drug combinations | Clinical investigators, regulators, statisticians | Opinion-based suggestions of 2 authors | The University of Ottawa and University of California San Diego Moores Cancer Centre (North America) | None | Oncology | No | Implicit | Selection | |
| The Association of the British Pharmaceutical Industry, 2018 (Self-published web-based document) (38) | Guidance document | To outline the framework within which Phase I research is conducted | Phase I research sponsors and investigators, ethics committee, trial subjects | • Synthesis of regulatory standards documents based on EU legislation, and incorporation of previous ABPI Phase I trial guidelines • Input from ABPI Experimental Medicine Expert Network and stakeholders from industry, regulators and professional organisations | ABPI (UK) | EMA, MHRA | General | No | Implicit | Selection; Stratification; Outcome assessment | |
| Vujanić et al., 2009 (Cancer) (39) | Research paper | To retrospectively examine the extent and quality of pathology material submitted for central pathology review (CPR) in international and national renal tumour trials to determine whether there were associations with improving the quality of material submitted for review and whether more widespread introduction of rapid CPR for all cases might be beneficial | Clinical trialists, pathologists, trial quality assurance managers involved in clinical trials of rare tumours | Conclusions from primary study, based on data from six previous multicentre paediatric renal tumours clinical trials from 1980 to 2007 | Cardiff University, Karolinska Institute, Royal Manchester Children's Hospital, Great Ormond Street Hospital for Children (UK, Sweden) | None | Oncology | No | Implicit | Selection; Stratification; Outcome assessment | |
| Yeung et al., 2016 (Nature Reviews Rheumatology) (40) | Discussion paper | To emphasize the need for standardisation of biospecimen collection and assay performance and the implementation of best practices in translational research in paediatric rheumatology, presented in the context of lessons learned from international biorepository networks | Biorepository personnel, collaborators in translational research networks, ethics committee, collaborative digital infrastructure information technology team, biobanking research community, scientific journal editors | Opinion-based suggestions from 6 authors | University of Toronto, Stanford University, Duke-National University of Singapore, University Medical Centre Utrecht, University College London (International representation from Canada, USA, Singapore, the Netherlands and UK) | None | Paediatric rheumatology | No | Implicit | Selection; Stratification; Outcome assessment | |
| ABPI: The Association of the British Pharmaceutical Industry; BIG: Breast International Group; BRISQ: Biospecimen Reporting for Improved Study Quality; CDP: Cancer Diagnosis Program; CM-Path: Cellular-Molecular Pathology; CTEP: Cancer Therapy Evaluation Program; EMA: European Medicines Agency; EORTC: European Organisation for Research and Treatment of Cancer; FDA: Food and Drug Administration; GBC: Group Banking Committee; GCP: Good Clinical Practice; IDSC: Investigational Drug Steering Committee; MHRA: Medicines and Healthcare products Regulatory Agency; NABCG: North American Breast Cancer Group; NCI: National Cancer Institute; NCRI: National Cancer Research Institute; NIH: National Institute of Health; NSCLC: Non-small-cell lung cancer; SOPs: Standard Operating Procedures | | | | | | | | | | | |

**Table S2.** Quality assessment of individual guidance resources

| **GRADE-CERQual domains** | **Methodological limitations** | **Adequacy of data** | | **Relevance** | **Coherence** | | |
| --- | --- | --- | --- | --- | --- | --- | --- |
| **AGREE-GRS domains**  **Guidance resources (Authors, Year)** | Process of development | Presentation style | Completeness of reporting | Clinical trial practice validity | Overall guideline quality | Overall assessment to recommend guideline for use in practice | Overall assessment to implement guideline in practice |
| Bossuyt et al., 2015 (1) | 83 | 100 | 50 | 92 | 83 | 100 | 100 |
| Dancey et al., 2010 (2) | 83 | 100 | 42 | 92 | 83 | 83 | 83 |
| Eberhard et al., 2008 (3) | 67 | 92 | 17 | 83 | 67 | 67 | 58 |
| European Medicines Agency, 2012 (4) | 33 | 67 | 8 | 100 | 50 | 100 | 100 |
| Hall et al., 2011 (5) | 58 | 92 | 17 | 100 | 58 | 83 | 83 |
| Hall et al., 2014 (6) | 75 | 100 | 42 | 100 | 83 | 92 | 92 |
| LaBaer, 2012 (7) | 33 | 50 | 25 | 75 | 42 | 50 | 50 |
| LaBaer et al.,  2018 (8) | 50 | 50 | 33 | 83 | 50 | 67 | 67 |
| Leyland-Jones et al., 2008 (9) | 100 | 92 | 67 | 100 | 92 | 100 | 100 |
| Loi et al., 2011 (10) | 67 | 75 | 42 | 92 | 58 | 50 | 50 |
| LoRusso et al., 2010 (11) | 67 | 92 | 17 | 92 | 67 | 83 | 83 |
| Maddocks et al., 2017 (12) | 58 | 75 | 42 | 83 | 58 | 58 | 58 |
| Makhlouf et al., 2019 (13) | 75 | 92 | 33 | 92 | 75 | 92 | 92 |
| Marton & Weiner,  2013 (14) | 33 | 92 | 33 | 92 | 67 | 83 | 83 |
| McShane & Hayes,  2012 (15) | 33 | 92 | 42 | 100 | 67 | 100 | 100 |
| McShane et al., 2013 (16) | 58 | 92 | 25 | 92 | 67 | 92 | 92 |
| Medicine and Healthcare products Regulatory Agency,  2009 (17) | 50 | 75 | 33 | 100 | 67 | 100 | 100 |
| Moore et al., 2011 (18) | 83 | 83 | 58 | 100 | 83 | 100 | 100 |
| Moorthy, 2018 (19) | 42 | 42 | 25 | 83 | 42 | 50 | 50 |
| Mroz et al., 2013 (20) | 50 | 33 | 42 | 83 | 50 | 67 | 67 |
| Nagtegaal et al., 2000 (21) | 92 | 75 | 92 | 92 | 83 | 92 | 92 |
| Nagtegaal et al., 2014 (22) | 50 | 92 | 50 | 92 | 67 | 83 | 83 |
| National Cancer Institute, 2016 (23) | 67 | 100 | 67 | 100 | 83 | 92 | 92 |
| Park et al., 2017 (24) | 92 | 83 | 92 | 92 | 83 | 100 | 100 |
| Pell et al., 2019 (25) | 67 | 67 | 67 | 83 | 67 | 83 | 83 |
| Peppercorn et al.,  2010 (26) | 50 | 75 | 50 | 100 | 67 | 92 | 92 |
| Pollack et al., 2003 (27) | 92 | 67 | 92 | 100 | 92 | 92 | 92 |
| Provenzano et al., 2015 (28) | 92 | 83 | 83 | 100 | 92 | 100 | 100 |
| Rees et al., 2019 (29) | 67 | 100 | 42 | 100 | 75 | 100 | 100 |
| Ricci & Franc, 2008 (30) (30) | 33 | 75 | 42 | 92 | 58 | 92 | 92 |
| Rimm et al., 2011 (31) | 67 | 83 | 42 | 92 | 67 | 92 | 92 |
| Robinson et al., 2019 (32) | 75 | 92 | 67 | 100 | 83 | 100 | 100 |
| Salgado et al., 2015 (33) | 100 | 100 | 83 | 100 | 100 | 100 | 100 |
| Sarzotti-Kelsoe et al., 2009 (34) | 83 | 100 | 92 | 100 | 92 | 92 | 92 |
| Scher et al., 2016 (35) | 92 | 100 | 83 | 100 | 92 | 92 | 92 |
| Spurgeon et al., 2017 (36) | 42 | 83 | 0 | 67 | 50 | 75 | 75 |
| Stewart & Kurzrock, 2013 (37) | 50 | 89 | 50 | 89 | 67 | 72 | 72 |
| The Association of the British Pharmaceutical Industry, 2018 (38) | 58 | 100 | 42 | 100 | 67 | 100 | 100 |
| Vujanić et al., 2009 (39) | 67 | 75 | 92 | 92 | 75 | 92 | 92 |
| Yeung et al., 2016 (40) | 42 | 42 | 25 | 75 | 42 | 67 | 67 |
| Scores for each AGREE-GRS domain on a 7-point scale are scaled to be calculated out of 100%, based on calculation methods from the AGREE-II User’s Manual(41). Scores of 33% or less are considered low quality (red), scores from 34% to 66% are considered moderate quality (yellow), scores of 67% or more are considered high quality (green). | | | | | | | |

**Table S3.** Clinical specialty-specific recommendations for biospecimen collection, processing, and histopathological assessment in clinical trials

| **Clinical specialty** | **Specific methods suggested** | **Number of resources** | **(%)** | **Citations** |
| --- | --- | --- | --- | --- |
| Breast cancer | Guidelines for assessment of tumour-infiltrating lymphocytes in breast cancer | 1 | 2.5 | (33) |
|  | Standardised methods for evaluation of post-neoadjuvant breast cancer specimens | 2 | 5 | (1,28) |
|  | Standardisation of biospecimen collection methods for breast cancer clinical trials | 2 | 5 | (9,10) |
|  |  |  |  |  |
| Non-small cell lung cancer | Standardisation of specimen preparation and molecular assays for evaluation of EGFR biomarker in lung cancer clinical trials | 1 | 2.5 | (3) |
|  |  |  |  |  |
| Paediatric neuroblastoma | Criteria for pathological complete response in neuroblastoma trials | 1 | 2.5 | (24) |
|  |  |  |  |  |
| Paediatric rheumatology | Standardisation of biological sample collection and assay performance in translational rheumatology | 1 | 2.5 | (40) |
|  |  |  |  |  |
| Prostate cancer | Recommendations for histology reporting in baseline patient assessment and response to treatment | 1 | 2.5 | (35) |
|  |  |  |  |  |
| Lymphoma | Criteria for biomarker evaluation and standardised evaluation methods for outcome assessment including central pathology review to confirm diagnosis | 2 | 5 | (12,36) |

**Table S4.** Meta-aggregative synthesis flowchart of pathology-specific recommendations according to different stages of the clinical trial process.

| **Clinical trial stage** | **Recommendation statements summarised from each individual resource** | **Quality of Statements** (AGREE-GRS scores mapped onto GRADE-CERQual domains) | | | | **Category** | **Synthesised recommendations across resources** |
| --- | --- | --- | --- | --- | --- | --- | --- |
|  |  | Methodological limitations | Adequacy | Relevance | Coherence |  |  |
| **Pre-analytical phase** | The clinical trial writing and management committees should include a pathologist who would be responsible for any biomarker use and other pathology-related issues such as guidelines, training, eligibility, stratification, quality control and treatment evaluation. (22) | 50 | 71 | 92 | 78 | Multidisciplinary collaboration and consultation with experts from diverse backgrounds in the design and setup of a clinical trial | **R1:** The responsibilities and level of involvement in clinical trial work should be agreed among all multidisciplinary collaborators and formally documented prior to trial opening. Input from pathologists and other relevant personnel with technical laboratory and statistical expertise and experience should be sought during the development of trial protocol, trial design and implementation, in particular, for justifying the use of biospecimens and/or a specific biomarker and associated analytical assays in the clinical trial, as well as reaching a consensus on prespecified definitions of pathology-related parameters when interpreting findings for trial inclusion or risk stratification. |
|  | Pathologists should be involved in the design and delivery of all trials to ensure maximum data collection and translational opportunities. (29) | 67 | 71 | 100 | 92 |  |  |
|  | The formulation of trial standard operating procedures should receive input from a pathology working group. Clinical trial protocols should define acceptable scanning platforms, file formats and scanning resolution. (25) | 67 | 67 | 83 | 78 |  |  |
|  | When designing the trial, there should be a formation of a ‘Pathology Working Group’ to oversee the pathology aspects of a study. (32) | 75 | 79 | 100 | 94 |  |  |
|  | Expertise, involvement and feedback from experts from many diverse disciplines should be initiated at the early stages of concept development and at various stages of protocol development to integrate collection of human biological materials into the timelines of the setup of a clinical trial. (5) | 58 | 54 | 100 | 75 |  |  |
|  | There should be transparent communication and collaboration among the trialists, medical professionals, pathology departments, and biobanks. (13) | 75 | 63 | 92 | 86 |  |  |
|  | In clinical research, pathologists should be in charge of pathology-related issues including methods for biospecimens storage, transportation, collection and processing. (23) | 67 | 83 | 100 | 89 |  |  |
|  | When designing the trial, the following should be considered: - estimates for uncertainty of a clinical trial test; - clear definitions for pathological complete response; - methods to ensure reproducibility and consistency of scoring and reporting pathology laboratory tests including pathologist calibration and achieving consensus on what is considered background staining; - account for the effect of diagnostic drift over duration of the trial on data analysis, when new disease classifications are published. (32) | 75 | 79 | 100 | 94 | Involvement of specific personnel with technical laboratory expertise for pathology-based parameters during the planning and designing of the trial |  |
|  | The rationale for sample size should be given of why a certain number of specimens should be collected and what specified effect size the study is designed to detect. There should be early consideration of the expected failure rate of the assay because assay failure rate can impact on the sample size (patient numbers) to enter the trial, make treatment decisions, and subsequently impacts the statistical design and power calculations. A key parameter that impacts on the sample size estimation is the frequency of occurrence of the biomarker, which can greatly influence the feasibility of accessing the required number of specimens to perform the work. (5) | 58 | 54 | 100 | 75 |  |  |
| **Pre-analytical phase** | Clinical trial proposals should include the following:  - feasibility of the study in terms of number of patients and specimens required to achieve adequate statistical power; - investigators’ level of expertise with proposed biomarkers and assays. (2) | 83 | 71 | 92 | 83 |  |  |
|  | Prior to trial opening: - sponsor should conduct a site evaluation to consider the site’s capabilities to meet the demands of a protocol, including any biomarker methodologies or sample acquisition analysis; - laboratory parameters should be prespecified, including the minimum dataset and minimum number of subjects required. (38) | 58 | 71 | 100 | 89 |  |  |
|  | Research plans should include: - Use of standardized, validated research biomarker assay. Reagents within assays should be fit-for-purpose and quality-controlled; - Statistical evaluation showing how the study question can be addressed with the samples available. (23) | 67 | 83 | 100 | 89 |  |  |
|  | Any study using research biopsies must be well designed to address the scientific question with a strong justification for the rationale of a biopsy for correlative end points. (26) | 50 | 63 | 100 | 83 | Rationale for use of biospecimens and/or specific biomarker and associated analytical assays in the clinical trial |  |
|  | Clinical trial protocol should provide a detailed description of all end-point measurements. The research protocol should be conceived to allow for long-term sample storage for the purpose of future research in order to maximize the potential value of the samples. (30) | 33 | 58 | 92 | 81 |  |  |
|  | The role and rationale of the use of the biomarker assay should be clearly stated and scientifically sound. Each biomarker study requires robust, validated assays that are fit for the intended purpose. The degree of analytical validity required for the assay must also be established. (5) | 58 | 54 | 100 | 75 |  |  |
|  | The Clinical Trials Design Taskforce of the National Cancer Institute Investigational Drug Steering Committee recommends that the rationale for inclusion of any biospecimens and associated biomarkers into phase I clinical trials should be justified. (11) | 67 | 54 | 92 | 78 |  |  |
|  | The rationale for tissue block submission and the scientific rigor of trials should be discussed with pathologists. Trialists should be transparent regarding the rationale and justification for the requested block submission. (13) | 75 | 63 | 92 | 86 |  |  |
|  | Clinical trial proposals should include a rationale for biomarker utility (eg. determining patient eligibility, stratification and/or outcome assessment) and appropriateness for the study, and the validity of the associated assay. (2) | 83 | 71 | 92 | 83 |  |  |
|  | To stratify patients reliably and evaluate trial outcomes clearly, methods of quantifying tumour content should be established prior to the start of the study, in addition to appropriate selection of biomarker tests with associated analytical parameters specifically validated for the specified disease. (19) | 42 | 33 | 83 | 47 |  |  |
| **Pre-analytical phase** | The clinical trial sponsor should ensure that pathologists and laboratory staff involved in clinical trial work should:  - undertake basic Good Clinical Practice training and understand the basic concepts involved in clinical trials research and clinical trial governance, - receive additional Good Clinical Laboratory Practice training in translational laboratory analysis of trial samples where appropriate, - pathologists should participate in specialist external quality assurance schemes appropriate to their field of clinical research involvement. (29) | 67 | 71 | 100 | 92 | Proper accreditation and provision of training for all personnel undertaking any aspect of clinical trial work | **R2:** All personnel undertaking any aspect of clinical trial work should have proper accreditation, sufficient funding and training corresponding to their involvement and role in the clinical trial. |
|  | When designing the trial, there should be appropriate training and accreditation for pathologists commensurate with role in clinical trial. (32) | 75 | 79 | 100 | 94 |  |  |
|  | The trial sponsor should ensure that all personnel (including laboratory and support staff) involved in a clinical trial have the appropriate experience or training to perform their job and for in-study protocol requirements. (34) | 83 | 96 | 100 | 92 |  |  |
|  | Participating pathologists should be appropriately trained before and during the trial in order to achieve a better accuracy and completeness of pathology data in clinical trials. (21) | 92 | 83 | 92 | 89 |  |  |
|  | Laboratory staff handling clinical trial specimens should receive Good Clinical Practice training, and have adequate experience and competency to follow laboratory’s standard operating procedures. (38) | 58 | 71 | 100 | 89 |  |  |
|  | All personnel involved in biospecimen resource management and use should be aware of purpose of biospecimen resource, regardless of their background discipline. If involved in specimen collection, they should be specifically trained with periodic retraining to adhere to standard operating procedures. (23) | 67 | 83 | 100 | 89 |  |  |
|  | If digital pathology and image analysis is used in clinical trials, participating pathologists should be specifically trained in digital image reporting. The costing of the pathologist and their training should be factored into the trial business plan. (25) | 67 | 67 | 83 | 78 | Appropriate funding allocation for materials, expertise and associated logistical procedures |  |
|  | At trial inception, the following considerations should be made: - incorporating cost of tissue collection or companion study into trial grant application; - funding for pathologists according to degree of trial involvement. (32) | 75 | 79 | 100 | 94 |  |  |
|  | When evaluating clinical trial proposals, funding should be prioritised for investigational imaging procedures, collection and storage of biospecimens. (2) | 83 | 71 | 92 | 83 |  |  |
| **Pre-analytical phase** | Pathology laboratories participating in clinical trials should be accredited and quality assured with an external quality assurance programme. (22) | 50 | 71 | 92 | 78 | Selection of laboratory site with suitable facilities to carry out trial work | **R3:** The laboratory site selected to carry out trial work should have:  (i) appropriate accreditation, with practices of laboratory management and operations complying with standards of regular external quality assurance schemes;  (ii) capacity to adhere to trial-specific standard operating procedures in the testing platforms, and preparation and storage of sample; and  (iii) suitable facilities required for the trial (e.g. accredited digital pathology platforms). |
|  | It is preferable to select an appropriately accredited laboratory with digital pathology facilities to participate in the trial. (25) | 67 | 67 | 83 | 78 |  |  |
|  | When designing a multicentre trial, the pre-specified site(s) of biomarker testing should be an ISO15189:2012-accredited and externally quality assured laboratories. (32) | 75 | 79 | 100 | 94 |  |  |
|  | Clinical laboratories participating in clinical trials should be externally accredited and meet Good Clinical Laboratory Practice compliance as the minimal requirement, to increase adherence to standardized practices and procedures, optimize laboratory operations, and to improve reproducibility and reliability of results, thereby enhancing data quality, while ensuring the safety of research participants. (34) | 83 | 96 | 100 | 92 |  |  |
|  | Laboratory should have external accreditation and participate in continual improvement schemes. (38) | 58 | 71 | 100 | 89 |  |  |
|  | When designing the trial, there should be detailed laboratory standard operating procedures for standardisation of block selection, sectioning protocols, testing platforms, and thresholds for positive results, to ensure consistent assessment of patient specimens across trial sites. (32) | 75 | 79 | 100 | 94 | Standardisation of laboratory pre-analytical processes |  |
|  | Trial pathology protocol should include guidelines on the standardisation of tissue handling and processing procedures, as well as proformas to ensure consistent data collection and complete datasets. (22) | 50 | 71 | 92 | 78 | Standardisation of sample collection and handling procedures |  |
|  | Prospective planning of biospecimen collection should be clearly written (specific preselected patient population, type of material, amount/volume, time point, etc) in the protocol, in order to allow harmonization of the collection and handling techniques, and planning for logistical and financial support, and integration with other aspects of the protocol. Logistics and handling protocols for biospecimen collection detailing the biospecimens shipment conditions and frequency, countries and sites involved, and requirements of the biomarker assay turnaround time, should be developed and distributed prospectively to the participating sites prior to biospecimen collection. (5) | 58 | 54 | 100 | 75 |  |  |
|  | All sample collection, handling, processing and storage should be done under predefined standard operating procedures. (7) | 33 | 38 | 75 | 47 |  |  |
|  | Biospecimens resources should: - develop standardised protocols for specimen storage, handling, transfer to maintain quality of specimens; - have a robust and encrypted informatics management system for backups and disaster recovery; - develop quality assurance policies to ensure efficient operation of the resource and safeguard against circumstances that could affect the scientific rigour of the research. (23) | 67 | 83 | 100 | 89 | Standardisation of biospecimens management procedures |  |
| **Analytical phase** | Digital pathology enables simultaneous case review, thereby identifying any discordant diagnostic parameters. (25) | 67 | 67 | 83 | 78 | Digital pathology has a role in pathology review which can improve diagnostic concordance rates | **R4:** Plans to ensure the completeness and accuracy of pathology-related datasets in clinical trials should be clearly documented and address the following:  (i) a system for prospective rapid real-time central pathology review or double reporting with consensus to achieve uniformity in diagnosis;  (ii) use of standardised digital pathology platforms where appropriate;  (iii) data quality review by a trained pathology quality manager or review committee to ensure adherence to standardised pathology examination and interpretation procedures;  (iv) regular analytical audits of internal testing platforms and assay performance testing, and validation of inter-pathologist reproducibility and interlaboratory repeatability analysis. |
|  | Digital pathology should be employed to streamline the central pathology review process in clinical trials, since it has been well-established that central pathology review helps improve concordance rates between pathologists. (20) | 50 | 38 | 83 | 61 |  |  |
|  | When central pathology review is performed as rapid real-time review, especially in rare tumours, there is a marked reduction of diagnostic discordances. As the central pathology review results would be communicated back to the institutional team prior to treatment implementation, achieving uniformity of diagnosis would help improve the quality of data for analysis. (39) | 67 | 83 | 92 | 86 | Central pathology review, if performed prospectively or as rapid real-time review, can improve diagnostic accuracy |  |
|  | Prospective central review ensures adherence to guidelines based on contemporary diagnostic classification systems, thus improving diagnostic accuracy and helps to confirm histological eligibility while minimizing inclusion of discordant cases. (27) | 92 | 79 | 100 | 92 |  |  |
|  | When designing the trial, the following should be considered: - systems for undertaking of pathological scoring and reporting to be blinded to treatment allocation and clinical outcomes; - double reporting and/or central pathology review of cases especially prior to final data analysis and trial publication. (32) | 75 | 79 | 100 | 94 | Data monitoring and validation (Improving the accuracy of pathology-related datasets directly contributing to analytical data for clinical trial) |  |
|  | The accuracy and completeness of pathology data in clinical trials can be improved by: - Standardisation of pathologic examination procedures; - Consecutive rounds of quality control by a trained pathology quality manager and a peer review committee. (21) | 92 | 83 | 92 | 89 |  |  |
|  | To minimize intra-reviewer bias, for study designs using a historical control group, the histology of both populations should be reviewed by the same individual based on the same review criteria, even as the inclusion criteria evolve over time. (27) | 92 | 79 | 100 | 92 |  |  |
|  | Transparent standards are encouraged in image research practice, including the diagnostic platform and image source code used, image analysis algorithms and reproducibility validation. In particular, there should be clearly documented quality control measures and validation steps in image analysis, including algorithm validation, measures of inter-pathologist reproducibility, and interlaboratory repeatability analysis. (25) | 67 | 67 | 83 | 78 | Quality assessment and auditing of laboratory-based procedures |  |
|  | Best practices for laboratory procedures within clinical trials include regular internal quality assessment audits, regular evaluation of assay performance and auditing test results against a pre-specified laboratory range with a reporting mechanism in place for identified outliers, and the use of a generalised proficiency testing program where applicable for validation and demonstrating fit-for-purpose methods for safety and endpoint analyses in clinical trials. (34) | 83 | 96 | 100 | 92 |  |  |
|  | Quality assurance and quality control programs can be implemented at different levels, that is, to check preanalytical conditions, to assess assay performance and reproducibility, and to analyse, interpret, and report assay results. (5) | 58 | 54 | 100 | 75 |  |  |
| **Post-analytical phase** | All standard operating procedures for sample collection, handling, processing and storage should be registered on a common and shared database. (7) | 33 | 38 | 75 | 47 | Data sharing of biospecimens preparation standard operating procedures | **R5:** The pathology-relevant aspects of clinical trials should be transparently reported according to the BRISQ (Biospecimen Reporting for Improved Study Quality) checklist and REMARK (REporting recommendations for tumour MARKer prognostic studies) criteria where appropriate, to include specific details relevant to biospecimen procurement, type, anatomical site, associated patients' clinical details, as well as protocols for tissue preparation, preservation and biomarker staining parameters. These standard operating procedures for the management of biospecimens during the clinical trial should be registered on a publicly accessible database, with digital location cited in the research publication. |
|  | The process of collection and preparation of tissue samples should be transparently reported in all relevant research publications, citing the digital location of sample preparation standard operating procedures and protocols where appropriate. Biorepositories should publish their biospecimen processing protocols and standard operating procedures in a publicly accessible database. (8) | 50 | 42 | 83 | 61 |  |  |
|  | Complete and transparent reporting of studies involving the use of prognostic and predictive tumour markers can be improved by: - adhering to relevant elements of the BRISQ (Biospecimen Reporting for Improved Study Quality) and CONSORT (Consolidated Standards Of Reporting Trials) guidelines with particular emphasis on the details of biospecimens and marker assays; - explicitly reporting all markers and end points that were examined in the study, stating any prespecified hypotheses and comprehensively describing all analytical methods used to produce the results including measures of uncertainty and estimated marker effects before and after adjustment of clinical and pathologic variables, in accordance with the REMARK (REporting recommendations for tumour MARKer prognostic studies) criteria. (15) | 33 | 67 | 100 | 89 | Transparent reporting of studies involving the use of biospecimens and biomarkers |  |
|  | The BRISQ (Biospecimen Reporting for Improved Study Quality) committee recommends that for studies involving the use of biospecimens, details relevant to pathology-related parameters should be transparently reported in the publication, including: - any formal certification or accreditation of the laboratory undertaking analysis of the biospecimen for the study; - the rationale for the chosen preservative parameters; - procedures and fixatives used for biospecimen collection, handling, preparation, storage (any standard operating procedures or protocols should be referenced and made publicly accessible); - type and anatomical site of the biospecimen; - the relation of the biospecimen to pertinent clinical diagnosis and patient characteristics; - the composition and pathology within the biospecimen. (18) | 83 | 71 | 100 | 94 |  |  |
| **Across all trial phases** | There should be policies or standard operating procedures in place to ensure that biospecimens should be completely anonymized from the original study subject. (30) | 33 | 58 | 92 | 81 | Confidentiality and data protection in the management of biospecimens | **R6:** Laboratories and biorepositories should have policies and procedures with secure informatics systems in place to minimise risks of harm to participants and to protect the confidentiality and data of participants, including anonymising collected biospecimens, tracking the movement of biospecimens within and across sites, and ensuring consent for biospecimen retention is valid prior to storage. |
|  | Informatics systems should be specifically designed to store data securely and to register and track samples. All procedures related to sample acquisition intended for genomic research should be accompanied by guidelines or formal standard operating procedures to ensure the quality and integrity of the samples and related data collected for these purposes. (30) | 33 | 58 | 92 | 81 |  |  |
|  | In terms of biobanking for future use, there should be processes in place to allow for tracking timelines for sample destruction. (30) | 33 | 58 | 92 | 81 |  |  |
|  | For the management of tissue blocks, pathology departments should be transparent with a written policy governing the release of blocks for clinical trials and biobanks should be transparent regarding their policies for block retention and return. (13) | 75 | 63 | 92 | 86 |  |  |
|  | Biospecimens resources should: - develop a robust unique identifier system that links participants’ information to the biospecimen confidentially and securely, and should also endure all storage conditions; - document their policies for retention of biospecimens and the protection of participants’ privacy and confidentiality of associated clinical data (including genetic data). (23) | 67 | 83 | 100 | 89 |  |  |
|  | Any study using research biopsies must be well designed to obtain the biopsy with minimal possible risk to study participants with minimal number of attempts. (26) | 50 | 63 | 100 | 83 | Ethical considerations around specimen collection and retention |  |
|  | In terms of biobanking for future use, study participants should be given the opportunity to agree separately to the storage of samples for future research. (30) | 33 | 58 | 92 | 81 |  |  |
| **Across all trial phases** | Before a specimen is obtained for research, participants should be fully informed of the risks, rationale, and requirements of the study, as well as of treatment alternatives. (26) | 50 | 63 | 100 | 83 | Key items to be included in patient information sheets and consent forms in trials | **R7:** Patient information sheets and consent forms should be reviewed by pathologists and should include information pertaining to the rationale of the use of biospecimen within the context of the clinical trial; the risks and benefits involved; how data will be analysed, stored, transferred between institutions, and shared with their healthcare providers; the details of biospecimen collection (type, frequency, volume or size of sample); and specimen retention policies for future research. In studies involving genomics or genetics, participants should specifically be counselled on the implications of a positive result on themselves and their relatives. |
|  | Informed consent document should include: - intended scope of use of samples - plans for duration of retention of samples and specifications for future use; - details for sample collection, including the number, volume or size of the sample and the timing of sampling. (30) | 33 | 58 | 92 | 81 |  |  |
|  | Patient information sheets and consent forms in trials should: - be reviewed by pathologists, if genetic studies are performed, to ensure that participants understand the implications of a positive result; - seek enduring consent if specimens are retained beyond the end of the trial. (32) | 75 | 79 | 100 | 94 |  |  |
|  | Key items that should be mentioned in the patient information sheet/ informed consent include what additional intervention or burden the patient may experience by participating in the biospecimens collection, if biospecimens may be shared with third parties for scientific research collaborations, and that biospecimens may be transferred out of the patients’ own country. (5) | 58 | 54 | 100 | 75 |  |  |
|  | Informed consent document should include biospecimen release policies for research and explain the benefits and possible risks when a site sends a block to a clinical trials biobank. Medical professionals should explain to patients the processes and policies governing disposition of their biospecimens and clearly communicate the pros and cons of tissue block submission for clinical trials with their patients. (13) | 75 | 63 | 92 | 86 |  |  |
|  | Informed consent documentation should include: - any tissue samples to be collected; - explicit consent for long-term storage if specimen is to be used for future research. (38) | 58 | 71 | 100 | 89 |  |  |
|  | Informed consent document should include: - a clear description of the operation of the biospecimen resource; - conditions under which samples and data will be released to recipient investigators; - policies pertaining to retention of biospecimens, privacy protection of research participants and confidentiality of data; - the nature and purpose of the research; - the types of data collected and how the data will be used and stored; - the benefits and risks as a research participant; - information on the types of genetic analysis that will be conducted and the consequences of DNA typing and associated anticipated risks posed to the research participant (for genetics or genomics-related research); - how biospecimens and/or data may be used by private or for-profit entities and the possibility of research leading to future development of commercial products, where applicable; - whether individual or aggregate research results will be released to the participant, the participant’s healthcare provider, or the participant’s family and, if so, the mechanism for communicating such results. (23) | 67 | 83 | 100 | 89 |  |  |

**Table S5.** GRADE-CERQual Evidence Profile.

| **Summary of review finding** (Synthesised recommendations) | **Resources contributing to review finding** | **Methodological limitations** | **Adequacy** | **Relevance** | **Coherence** | **GRADE-CERQual assessment of confidence in the evidence** | **Explanation of GRADE-CERQual assessment** |
| --- | --- | --- | --- | --- | --- | --- | --- |
| **R1:** The responsibilities and level of involvement in clinical trial work should be agreed among all multidisciplinary collaborators and formally documented prior to trial opening. Input from pathologists and other relevant personnel with technical laboratory and statistical expertise and experience should be sought during the development of trial protocol, trial design and implementation, in particular, for justifying the use of biospecimens and/or a specific biomarker and associated analytical assays in the clinical trial, as well as reaching a consensus on prespecified definitions of pathology-related parameters when interpreting findings for trial inclusion or risk stratification. | (2,5,30,32,38,11,13,19,22,23,25,26,29) | Moderate methodological limitations  (2 resources (5,38) with minor limitations (multidisciplinary working group with unclear guideline development process) and 4 resources (19,22,26,30) with moderate methodological limitations (single author or few authors' opinions only)) | Moderate concerns  (5 resources (5,11,13,26,30) with minor concerns (thin data) and 1 resource (19) with serious concerns (extremely limited, thin data)) | No concerns | Very minor concerns   (minor concern about the fit between the data in 1 resource (19) and the review finding) | Moderate confidence | Moderate methodological limitations and moderate concerns regarding adequacy of data. |
| **R2:** All personnel undertaking any aspect of clinical trial work should have proper accreditation, sufficient funding and training corresponding to their involvement and role in the clinical trial. | (2,21,23,25,29,32,34,38) | Very minor methodological limitations  (only 1 resource (38) has minor methodological limitations (unclear guideline development process)) | No concerns | No concerns | No concerns | High confidence | No or very minor concerns regarding methodological limitations, adequacy of data, relevance and coherence. |
| **R3:** The laboratory site selected to carry out trial work should have:  (i) appropriate accreditation, with practices of laboratory management and operations complying with standards of regular external quality assurance schemes;  (ii) capacity to adhere to trial-specific standard operating procedures in the testing platforms, and preparation and storage of sample; and  (iii) suitable facilities required for the trial (e.g. accredited digital pathology platforms). | (5,7,22,23,25,32,34,38) | Moderate methodological limitations  (2 resources (5,38) with minor limitations (multidisciplinary working group with unclear guideline development process), 1 resource (22) with moderate limitations (few authors' opinions) and 1 resource (7) with serious methodological limitations (single author's opinions only - not all backed up by literature)) | Minor concerns  (2 resources (5,7) have minor concerns (thin data) but the other 6 resources together offer moderately rich data) | No concerns | Very minor concerns  (minor concern about the fit between the data in 1 resource (7) and the review finding) | Moderate confidence | Moderate methodological limitations and minor concerns regarding adequacy of data. |
| **R4:** Plans to ensure the completeness and accuracy of pathology-related datasets in clinical trials should be clearly documented and address the following:  (i) a system for prospective rapid real-time central pathology review or double reporting with consensus to achieve uniformity in diagnosis;  (ii) use of standardised digital pathology platforms where appropriate;  (iii) data quality review by a trained pathology quality manager or review committee to ensure adherence to standardised pathology examination and interpretation procedures;  (iv) regular analytical audits of internal testing platforms and assay performance testing, and validation of inter-pathologist reproducibility and interlaboratory repeatability analysis. | (5,20,21,25,27,32,34,39) | Very minor methodological limitations  (only 2 resources (5,20) have minor methodological limitations (unclear guideline development process)) | Minor concerns  (1 resource (5) with minor concerns (thin data) and 1 resource (20) with moderate concerns (limited thin data)) | No concerns | No concerns | High confidence | Minor concerns regarding adequacy of data. No or very minor concerns regarding methodological limitations, relevance and coherence. |
| **R5:** The pathology-relevant aspects of clinical trials should be transparently reported according to the BRISQ (Biospecimen Reporting for Improved Study Quality) checklist and REMARK (REporting recommendations for tumour MARKer prognostic studies) criteria where appropriate, to include specific details relevant to biospecimen procurement, type, anatomical site, associated patients' clinical details, as well as protocols for tissue preparation, preservation and biomarker staining parameters. These standard operating procedures for the management of biospecimens during the clinical trial should be registered on a publicly accessible database, with digital location cited in the research publication. | (7,8,15,18) | Serious methodological limitations  (2 resources with moderate limitations (recommendations based on few authors' opinions (8); literature review with unclear methodology (15)) and 1 resource (7) with serious methodological limitations (single author's opinions only - not all backed up by literature)) | Moderate concerns  (1 resource (7) with minor concerns (thin data) and 1 resource (8) with moderate concerns (very limited thin data)) | No concerns | Minor concerns  (minor concern about the fit between the data in 2 resources (7,8) and the review finding) | Low confidence | Serious methodological limitations and moderate concerns regarding adequacy of data. |
| **R6:** Laboratories and biorepositories should have policies and procedures with secure informatics systems in place to minimise risks of harm to participants and to protect the confidentiality and data of participants, including anonymising collected biospecimens, tracking the movement of biospecimens within and across sites, and ensuring consent for biospecimen retention is valid prior to storage. | (13,23,26,30) | Moderate methodological limitations  (2 resources (26,30) with moderate methodological limitations (single author or few authors' opinions only)) | Moderate concerns  (only 1 resource (23) offers rich data while the other 3 resources (13,26,30) have thin data (minor concerns)) | No concerns | No concerns | Moderate confidence | Moderate methodological limitations and moderate concerns regarding adequacy of data. |
| **R7:** Patient information sheets and consent forms should be reviewed by pathologists and should include information pertaining to the rationale of the use of biospecimen within the context of the clinical trial; the risks and benefits involved; how data will be analysed, stored, transferred between institutions, and shared with their healthcare providers; the details of biospecimen collection (type, frequency, volume or size of sample); and specimen retention policies for future research. In studies involving genomics or genetics, participants should specifically be counselled on the implications of a positive result on themselves and their relatives. | (5,13,23,26,30,32,38) | Moderate methodological limitations  (2 resources (5,38) with minor limitations (multidisciplinary working group with unclear guideline development process) and 2 resources (26,30) with moderate methodological limitations (single author or few authors' opinions only)) | Moderate concerns  (only 3 resources (23,32,38) together offer moderately rich data, the other 4 resources (5,13,26,30) have thin data (minor concerns)) | No concerns | No concerns | Moderate confidence | Moderate methodological limitations and moderate concerns regarding adequacy of data. |

**Table S6.** GRADE-CERQual Summary of Qualitative Findings.

| **Objective:** To identify, appraise and synthesise qualitative evidence of recommendations specific to cellular and molecular pathology input in clinical trials. **Population:** Individuals/ relevant stakeholders involved in the design, conduct and analysis of clinical trials requiring cellular and molecular pathology input. **Perspective/ Phenomena of Interest:** recommendations or guidelines specific to cellular and molecular pathology input, derived from published opinions and/or experiences of relevant stakeholders with expertise in pathology-related parameters in the clinical trial process. **Context:** the entire clinical trial process, from the design and conduct of the trial, to the analysis and dissemination of trial findings. **Resources included in synthesis**:  Perspective papers, literature reviews, primary research studies, guidance documents from research institute and research-based industry, letters to editors, book chapter. **Geographical/ organisational representation in guidance development of resources included in synthesis:**  USA (10 resources); UK (4 resources); Europe (4 resources); North America (2 resources); International (2 resources). | | | | | | | |
| --- | --- | --- | --- | --- | --- | --- | --- |
| **Aspect of clinical trial process** | **Summary of review finding** (Synthesised recommendations) | **Relevance to key clinical trial stakeholders** | **Relevance to current clinical trial checklists** | | **Resources contributing to review finding** | **CERQual assessment of confidence in the evidence** | **Explanation of CERQual assessment** |
|  |  |  | SPIRIT 2013 checklist sections and associated item numbers | CONSORT 2010 checklist sections and associated item numbers |  |  |  |
| Personnel involved in *a priori* planning (pathology and statistical expertise);  Rationale for use of biospecimen and/or biomarker | The responsibilities and level of involvement in clinical trial work should be agreed among all multidisciplinary collaborators and formally documented prior to trial opening. Input from pathologists and other relevant personnel with technical laboratory and statistical expertise and experience should be sought during the development of trial protocol, trial design and implementation, in particular, for justifying the use of biospecimens and/or a specific biomarker and associated analytical assays in the clinical trial, as well as reaching a consensus on prespecified definitions of pathology-related parameters when interpreting findings for trial inclusion or risk stratification. | Trial protocol writers, study sponsors, bioinformaticians, statisticians, clinicians from respective disciplines, other personnel in supporting roles. | Trial registration (2), Protocol (3), Roles and responsibilities (5).  Background and rationale (6), Objectives (7), Trial design (8), Eligibility criteria (10), Outcomes (12), Sample size (14), Statistical methods (20). | Registration (23), Protocol (24).  Background and objectives (2), Trial design (3), Participants (4), Outcomes (6), Sample size (7), Statistical methods (12). | (2,5,30,32,38,11,13,19,22,23,25,26,29) | Moderate confidence | Moderate methodological limitations and moderate concerns regarding adequacy of data. |
| Funding and training of individuals contributing to clinical trial work | All personnel undertaking any aspect of clinical trial work should have proper accreditation, sufficient funding and training corresponding to their involvement and role in the clinical trial. | Trial protocol writers, study sponsors, funding bodies, research regulatory agencies. | Trial registration (2), Protocol (3), Funding (4),  Roles and responsibilities (5),  Trial design (8) | Registration (23), Protocol (24), Trial design (3), Funding (25). | (2,21,23,25,29,32,34,38) | High confidence | No or very minor concerns regarding methodological limitations, adequacy of data, relevance and coherence. |
| Study setting and facilities | The laboratory site selected to carry out trial work should have:  (i) appropriate accreditation, with practices of laboratory management and operations complying with standards of regular external quality assurance schemes;  (ii) capacity to adhere to trial-specific standard operating procedures in the testing platforms, and preparation and storage of sample; and  (iii) suitable facilities required for the trial (eg. accredited digital pathology platforms). | Study sponsors, Chief investigator, trial protocol writers. | Trial registration (2), Protocol (3), Trial design (8), Study setting (9),  Data collection methods (18), Data management (19). | Trial design (3), Participants (4), Outcomes (6), Outcomes and estimation (17),  Ancillary analyses (18). | (5,7,22,23,25,32,34,38) | Moderate confidence | Moderate methodological limitations and minor concerns regarding adequacy of data. |
| Quality assurance and ensuring accuracy of pathology-related data | Plans to ensure the completeness and accuracy of pathology-related datasets in clinical trials should be clearly documented and address the following:  (i) a system for prospective rapid real-time central pathology review or double reporting with consensus to achieve uniformity in diagnosis;  (ii) use of standardised digital pathology platforms where appropriate;  (iii) data quality review by a trained pathology quality manager or review committee to ensure adherence to standardised pathology examination and interpretation procedures;  (iv) regular analytical audits of internal testing platforms and assay performance testing, and validation of inter-pathologist reproducibility and interlaboratory repeatability analysis. | Quality assurance team, Data monitoring committee | Eligibility criteria (10),  Outcomes (12),  Data management (19), Data monitoring (21), Auditing (23), Biological specimens (33). | Trial design (3), Participants (4), Outcomes (6), Blinding (11), Participant flow (13), Baseline data (15), Numbers analysed (16), Outcomes and estimation (17),  Ancillary analyses (18), Generalisability (21),  Interpretation (22). | (5,20,21,25,27,32,34,39) | High confidence | Minor concerns regarding adequacy of data. No or very minor concerns regarding methodological limitations, relevance and coherence. |
| Data sharing and transparent reporting | The pathology-relevant aspects of clinical trials should be transparently reported according to the BRISQ (Biospecimen Reporting for Improved Study Quality) checklist and REMARK (REporting recommendations for tumour MARKer prognostic studies) criteria where appropriate, to include specific details relevant to biospecimen procurement, type, anatomical site, associated patients' clinical details, as well as protocols for tissue preparation, preservation and biomarker staining parameters. These standard operating procedures for the management of biospecimens during the clinical trial should be registered on a publicly accessible database, with digital location cited in the research publication. | Study authors, Organisations supporting the trial (e.g. biorepositories and laboratory institutes), Journal editors | Access to data (29), Dissemination policy (31),  Biological specimens (33). | Ancillary analyses (18), Limitations (20),  Generalisability (21), Interpretation (22). | (7,8,15,18) | Low confidence | Serious methodological limitations and moderate concerns regarding adequacy of data. |
| Ethical considerations and maintaining confidentiality and data protection in the management of biospecimens | Laboratories and biorepositories should have policies and procedures with secure informatics systems in place to minimise risks of harm to participants and to protect the confidentiality and data of participants, including anonymising collected biospecimens, tracking the movement of biospecimens within and across sites, and ensuring consent for biospecimen retention is valid prior to storage. | Study participants, Patient advocates, Informaticians, Data management committee, Research ethics committee, Clinicians | Harms (22),  Research ethics approval (24), Confidentiality (27), Consent or assent (26), Ancillary and posttrial care (30), Biological specimens (33). | Harms (19) | (13,23,26,30) | Moderate confidence | Moderate methodological limitations and moderate concerns regarding adequacy of data. |
| Informed consent documentation | Patient information sheets and consent forms should be reviewed by pathologists and should include information pertaining to the rationale of the use of biospecimen within the context of the clinical trial; the risks and benefits involved; how data will be analysed, stored, transferred between institutions, and shared with their healthcare providers; the details of biospecimen collection (type, frequency, volume or size of sample); and specimen retention policies for future research. In studies involving genomics or genetics, participants should specifically be counselled on the implications of a positive result on themselves and their relatives. | Study participants, Patient advocates, Research ethics committee, Clinicians and researchers obtaining consent | Consent or assent (26), Informed consent materials (32), Biological specimens (33). | None | (5,13,23,26,30,32,38) | Moderate confidence | Moderate methodological limitations and moderate concerns regarding adequacy of data. |

**References:**

1. Bossuyt V, Provenzano E, Symmans WF, Boughey JC, Coles C, Curigliano G, et al. Recommendations for standardized pathological characterization of residual disease for neoadjuvant clinical trials of breast cancer by the BIG-NABCG collaboration. Ann Oncol Off J Eur Soc Med Oncol. 2015 Jul;26(7):1280–91.

2. Dancey JE, Dobbin KK, Groshen S, Jessup JM, Hruszkewycz AH, Koehler M, et al. Guidelines for the development and incorporation of biomarker studies in early clinical trials of novel agents. Clin Cancer Res. 2010;16(6):1745–55.

3. Eberhard DA, Giaccone G, Johnson BE. Biomarkers of response to epidermal growth factor receptor inhibitors in non-small-cell lung cancer working group: Standardization for use in the clinical trial setting. J Clin Oncol. 2008;26(6):983–94.

4. European Medicines Agency. Reflection paper for laboratories that perform the analysis or evaluation of clinical trial samples. EMEA Guid Doc. 2012;1–19.

5. Hall JA, Daidone MG, Peters GJ, Harbeck N, Lacombe D. Integrating collection of biospecimens in clinical trials: The approach of the european organization for research and treatment of cancer. Biopreserv Biobank. 2011;9(2):181–6.

6. Hall JA, Salgado R, Lively T, Sweep F, Schuh A. A risk-management approach for effective integration of biomarkers in clinical trials: perspectives of an NCI, NCRI, and EORTC working group. Lancet Oncol. 2014;15(4):e184-93.

7. LaBaer J. Improving international research with clinical specimens: 5 achievable objectives. J Proteome Res. 2012 Dec;11(12):5592–601.

8. LaBaer J, Miceli JF, Freedman LP. What’s in a sample? Increasing transparency in biospecimen procurement methods. Nat Methods. 2018 May 27;15(5):303–4.

9. Leyland-Jones BR, Ambrosone CB, Bartlett J, Ellis MJC, Enos RA, Raji A, et al. Recommendations for collection and handling of specimens from group breast cancer clinical trials. J Clin Oncol. 2008 Dec;26(34):5638–44.

10. Loi S, Symmans WF, Bartlett JMS, Fumagalli D, Van’t Veer L, Forbes JF, et al. Proposals for uniform collection of biospecimens from neoadjuvant breast cancer clinical trials: timing and specimen types. Lancet Oncol. 2011;12(12):1162–8.

11. LoRusso PM, Boerner SA, Seymour L. An overview of the optimal planning, design, and conduct of phase I studies of new therapeutics. Clin Cancer Res. 2010;16(6):1710–8.

12. Maddocks K, Barr PM, Cheson BD, Little RF, Baizer L, Kahl BS, et al. Recommendations for clinical trial development in follicular lymphoma. J Natl Cancer Inst. 2017;109(3):djw255.

13. Makhlouf H, Watson MA, Lankes HA, Weil C, Dickler M, Birrer M, et al. Toward Improving Practices for Submission of Diagnostic Tissue Blocks for National Cancer Institute Clinical Trials. Am J Clin Pathol. 2019 Oct 15;153(2):149–55.

14. Marton MJ, Weiner R. Practical guidance for implementing predictive biomarkers into early phase clinical studies. Biomed Res Int. 2013;(4):891391.

15. McShane LM, Hayes DF. Publication of tumor marker research results: the necessity for complete and transparent reporting. J Clin Oncol. 2012;30(34):4223–32.

16. McShane LM, Cavenagh MM, Lively TG, Eberhard DA, Bigbee WL, Williams PM, et al. Criteria for the use of omics-based predictors in clinical trials. Nature. 2013;502(7471):317–20.

17. Medicine and Healthcare products Regulatory Agency (MHRA). Guidance on the maintenance of regulatory compliance in laboratories that perform the analysis or evaluation of clinical trial samples. [Internet]. 2009. Available from: http://www.mhra.gov.uk/home/groups/is-insp/documents/websiteresources/con051910.pdf

18. Moore HM, Kelly AB, Jewell SD, McShane LM, Clark DP, Greenspan R, et al. Biospecimen Reporting for Improved Study Quality (BRISQ). J Proteome Res. 2011 Aug;10(8):3429–38.

19. Moorthy T. Targeted therapy: a potential oversight in trial protocol. Lancet Oncol. 2018;19(9):e439.

20. Mroz P, Parwani A V, Kulesza P. Central pathology review for phase III clinical trials: the enabling effect of virtual microscopy. Arch Pathol Lab Med. 2013 Apr;137(4):492–5.

21. Nagtegaal I, Klein Kranenbarg E, Hermans J, van de Velde CH, van Krieken J. Pathology data in the central databases of multicenter randomized trials need to be based on pathology reports and controlled by trained quality managers. J Clin Oncol. 2000;18(8):1771–9.

22. Nagtegaal ID, West NP, van Krieken JHJM, Quirke P. Pathology is a necessary and informative tool in oncology clinical trials. J Pathol. 2014;232(2):185–9.

23. National Cancer Institute. NCI Best Practices for Biospecimen Resources. 2016;1–81.

24. Park JR, Bagatell R, Cohn SL, Pearson AD, Villablanca JG, Berthold F, et al. Revisions to the International Neuroblastoma Response Criteria: A Consensus Statement From the National Cancer Institute Clinical Trials Planning Meeting. J Clin Oncol. 2017;35(22):2580–7.

25. Pell R, Oien K, Robinson M, Pitman H, Rajpoot N, Rittscher J, et al. The use of digital pathology and image analysis in clinical trials. J Pathol Clin Res. 2019 Apr 25;5(2):81–90.

26. Peppercorn J, Shapira I, Collyar D, Deshields T, Lin N, Krop I. Ethics of mandatory research biopsy for correlative end points within clinical trials in oncology. J Clin Oncol. 2010;28(15):2635–40.

27. Pollack IF, Boyett JM, Yates AJ, Burger PC, Gilles FH, Davis RL, et al. The influence of central review on outcome associations in childhood malignant gliomas: results from the CCG-945 experience. Neuro Oncol. 2003 Jul;5(3):197–207.

28. Provenzano E, Bossuyt V, Viale G, Cameron D, Badve S, Denkert C, et al. Standardization of pathologic evaluation and reporting of postneoadjuvant specimens in clinical trials of breast cancer: Recommendations from an international working group. Mod Pathol. 2015;28(9):1185–201.

29. Rees G, Salto-Tellez M, Lee J, Oien K, Verrill C, Freeman A, et al. Training and accreditation standards for pathologists undertaking clinical trial work. J Pathol Clin Res. 2019;5(2):100–7.

30. Ricci DS, Franc M. Implementation of pharmacogenomic sample collection in clinical trials. In: Pharmacogenomics and Personalized Medicine. Humana Press; 2008. p. 27–54.

31. Rimm DL, Nielsen TO, Jewell SD, Rohrer DC, Broadwater G, Waldman F, et al. Cancer and leukemia group B pathology committee guidelines for tissue microarray construction representing multicenter prospective clinical trial tissues. J Clin Oncol. 2011;29(16):2282–90.

32. Robinson M, James J, Thomas G, West N, Jones L, Lee J. Quality assurance guidance for scoring and reporting for pathologists and laboratories undertaking clinical trial work. J Pathol Clin Res. 2019;5(2):91–9.

33. Salgado R, Denkert C, Demaria S, Sirtaine N, Klauschen F, Pruneri G, et al. The evaluation of tumor-infiltrating lymphocytes (TILs) in breast cancer: recommendations by an International TILs Working Group 2014. Ann Oncol. 2015;26(2):259–71.

34. Sarzotti-Kelsoe M, Cox J, Cleland N, Denny T, Hural J, Needham L, et al. Evaluation and recommendations on good clinical laboratory practice guidelines for phase I-III clinical trials. PLoS Med. 2009;6(5):e1000067.

35. Scher HI, Morris MJ, Stadler WM, Higano C, Basch E, Fizazi K, et al. Trial Design and Objectives for Castration-Resistant Prostate Cancer: Updated Recommendations From the Prostate Cancer Clinical Trials Working Group 3. J Clin Oncol. 2016;34(12):1402–18.

36. Spurgeon SE, Till BG, Martin P, Goy AH, Dreyling MP, Gopal AK, et al. Recommendations for Clinical Trial Development in Mantle Cell Lymphoma. J Natl Cancer Inst. 2017;109(1).

37. Stewart DJ, Kurzrock R. Fool’s gold, lost treasures, and the randomized clinical trial. BMC Cancer. 2013;13:193.

38. The Association of the British Pharmaceutical Industry (ABPI). Guidelines for Phase I clinical trials [Internet]. 2018 [cited 2020 Apr 3]. Available from: https://www.abpi.org.uk/media/4992/guidelines-for-phase-i-clinical-trials-2018-edition-20180626.pdf

39. Vujanić GM, Sandstedt B, Kelsey A, Sebire NJ. Central pathology review in multicenter trials and studies: Lessons from the nephroblastoma trials. Cancer. 2009;115(9):1977–83.

40. Yeung R, Albani S, Feldman B, Mellins E, Prakken B. Enhancing translational research in paediatric rheumatology through standardization. Nat Rev Rheumatol. 2016;12(11):684–90.

41. AGREE. AGREE II User Manual [Internet]. 2017 [cited 2020 Jul 9]. p. 9. Available from: https://www.agreetrust.org/wp-content/uploads/2017/12/AGREE-II-Users-Manual-and-23-item-Instrument-2009-Update-2017.pdf
